# Stabilizing a Native Fold of Alpha-Synuclein with Short Helix-Constrained Peptides

**DOI:** 10.1021/jacsau.5c00694

**Published:** 2025-09-04

**Authors:** Richard M. Meade, Scott G. Allen, Amy J. Lopez, Christopher Williams, Iona Thomas-Wright, Rachel Heon-Roberts, Mara Carey-Wood, T. M. Simon Tang, Julia E. Sero, Vicky L. Hunt, Richard Wade-Martins, Matthew P. Crump, Jody M. Mason

**Affiliations:** † Department of Life Sciences, 1555 University of Bath, Claverton Down, Bath BA2 7AY, United Kingdom; ‡ School of Chemistry, 1980 University of Bristol, Cantock’s Close, Bristol BS8 1TS, United Kingdom; § Department of Physiology, Anatomy and Genetics, Oxford Parkinson’s Disease Centre, Oxford OX1 3QX, United Kingdom

**Keywords:** peptide, amyloid aggregation, lipid induced aggregation, lipid vesicles, Parkinson’s disease

## Abstract

Preventing the aggregation of α-synuclein (αS) into toxic oligomers and conformers is a major therapeutic goal in conditions such as Parkinson’s disease and Lewy body dementia. However, the large intracellular protein–protein interfaces within such aggregates make this a challenging target for small molecule approaches or biologics, which often lack cell permeability. Peptides occupy a suitable middle ground and are increasingly being explored as preventative treatments. We previously showed that the N-terminal lipid binding region (αS_1–25_) inhibits αS aggregation. Building on this, we designed a series of N- and C-terminal truncations to systematically reduce the peptide length, enabling a 56% downsizing (i.e., truncating 92% of the full-length αS protein), to identify the smallest functional unit capable of binding αS and potently blocking its aggregation and toxicity. We next introduced seven systematic i → i + 4 helix constraints to assess impact on (i) α-helicity, (ii) aggregation inhibition, (iii) serum stability, (iv) neuronal uptake, and (v) phenotypic rescue. This work maps key amphipathic features and identifies residues that are critical for αS engagement and inhibitory activity. The most effective helix-constrained peptide, αS_2–12_(L6), showed marked improvements across all metrics and represents a strong candidate for further therapeutic development.

## Graphical abstract

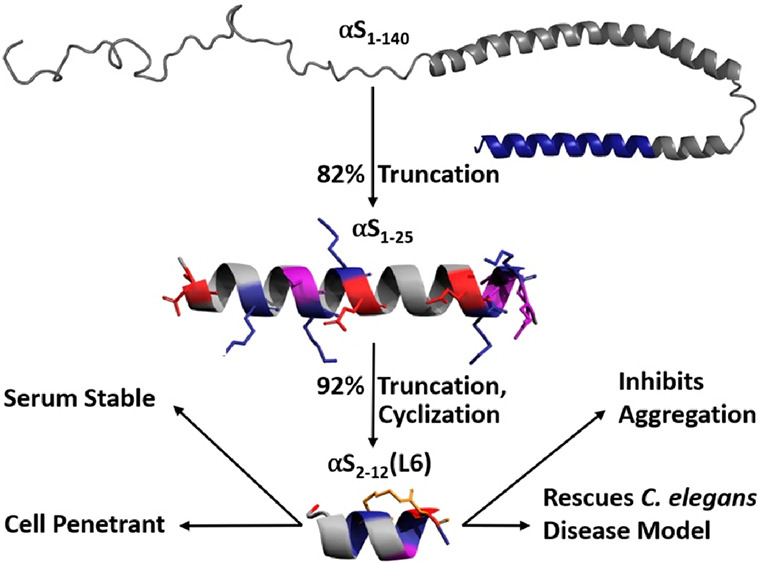

## Introduction

The misfolding and aggregation of alpha synuclein (αS) into toxic oligomers, fibrils, and Lewy bodies is a defining pathogenic hallmark of Parkinson’s disease (PD) and related synucleinopathies. Identifying molecules that selectively bind and neutralize αS assemblies has long been a key therapeutic goal.^1,5,6^ Lewy bodies accumulate in the cytoplasm of dopaminergic neurons in the *substantia nigra pars compacta* (*SNCA*), disrupting dopamine signaling, triggering neuronal death, and ultimately leading to the symptoms of PD. Extensive evidence links αS to PD, with single-point mutations^7–11^ and *SNCA* gene duplication^12^ or triplication^13^ accelerating pathology by several decades relative to sporadic onset.

In its native state, αS exists as an intrinsically disordered protein (IDP) that can aggregate into β-sheet-rich amyloid structures. However, αS can also undergo ‘folding upon binding’, transitioning from an IDP state to an α-helix-rich conformation upon lipidic interaction (Figure 1A). This stabilized helical form is closely linked to the native αS function of vesicle budding, accumulation, and fusion to the presynaptic membrane, thereby modulating synaptic transmission.^1,14^ αS folding and lipid interactions are central to its role in modulating vesicles involved in interneuronal dopamine transport.^2,15^ Early onset *SNCA* mutations are thought to destabilize this native αS structure.^16^ Here, we propose a strategy to prevent αS aggregation by stabilizing its functional, lipid-bound conformation. The N-terminal domain of αS populates an α-helical structure upon lipid binding^2^ (PDB_ID_ = 1XQ8), a finding we also demonstrated by solving the solution NMR structure of αS_1–25_ in 50% trifluoroethanol.^1^ Membrane recognition by this N-terminal region is essential for driving cooperative helix formation across the remainder of the protein.^17–20^

Extensive efforts to identify modulators of αS aggregation have yielded a range of candidates, including peptides,^21^ that influence misfolding and aggregation.^19,22–29^ In the broader peptide field, it is well established that conformational constraints can impose structural rigidity and stabilize defined secondary structures, often greatly enhancing the peptide-target affinity.^30,31^ Based on this principle, we sought to combine terminal truncation with helix-inducing constraints in a previously characterized helical peptide inhibitor of αS aggregation,^1^ aiming to shorten the peptide while maintaining or improving binding affinity. In addition to promoting binding, the constraint may functionally mimic lipid interactions by inducing helical structure and improving engagement with αS. Finally, because αS exists as a highly dynamic ensemble of oligomeric and conformational states, introducing a conformationally preorganized peptide offers the potential to selectively recognize and ultimately stabilize a specific nontoxic αS conformer, ideally its native, functional state.

Protein–protein interactions (PPIs) typically involve large, flat surfaces with multiple weak contact points and often lack the well-defined hydrophobic pockets required for traditional small molecules, making them notoriously difficult to drug.^32^ This challenge is particularly true of amyloids, which form through the acquisition of extended β-sheet-rich structures. Peptides offer several advantages over larger biologics, such as antibodies or proteins. They are uniquely suited to bind large, shallow, extended interfaces with high selectivity, while remaining small enough to penetrate biological membranes and access intracellular targets like αS. The therapeutic potential of peptide-based molecules has been widely recognized,^21,32–36^ and numerous strategies now exist to overcome traditional limitations such as poor stability, membrane permeability, or bioavailability. With advances in design and delivery, peptide-based drugs can now be made stable, cell/Blood-Brain-Barrier (BBB) permeable, and even orally bioavailable, with half-lives extending to days.^32,35,37^ The helix-constrained peptide approach employed here has yielded serum-stable molecules that selectively stabilize the native conformation of αS, effectively blocking downstream aggregation and toxicity. Biophysical and structural characterization confirms that these constrained peptides engage and stabilize αS_1–140_ in its functional, premisfolded state, intervening early to prevent the cascade of pathological aggregation.

## Results and Discussion

We previously showed that both full-length αS (Figure 1B) and its N-terminal segment, αS_1–25_ (Figure 1C), exist as intrinsically disordered random coils in isolation but adopt a highly α-helical structure (∼58% helicity) in the presence of 1,2-dimyristoyl-*sn*-glycero-3-phospho-l-serine (DMPS) lipid vesicles.^1^ DMPS is particularly well-suited for this role, as it constitutes a major phospholipid component (∼12%) of dopaminergic synaptic vesicles.^38^ With its negatively charged headgroup, DMPS promotes αS membrane binding and elevates the local αS concentration. These conditions are known to accelerate aggregation via a two-step nucleation process.^24,25,27,39^ Subsequently, we demonstrated that αS_1–25_ can inhibit αS aggregation in the presence of these lipid vesicles (Figure 1D).^1^ Here, we advance that work by (i) iteratively truncating αS_1–25_ to identify the minimal active sequence and key residues required for inhibitory function, and (ii) introducing all seven possible i → i + 4 helix-inducing constraints into the resulting αS_2–12_ sequence to improve efficacy. By determining which constraints enhance both helicity and inhibitory activity, we have defined the functional face of the helix required for αS binding, and the noninteracting face suitable for cross-linking or further modification.

**Figure 1:**
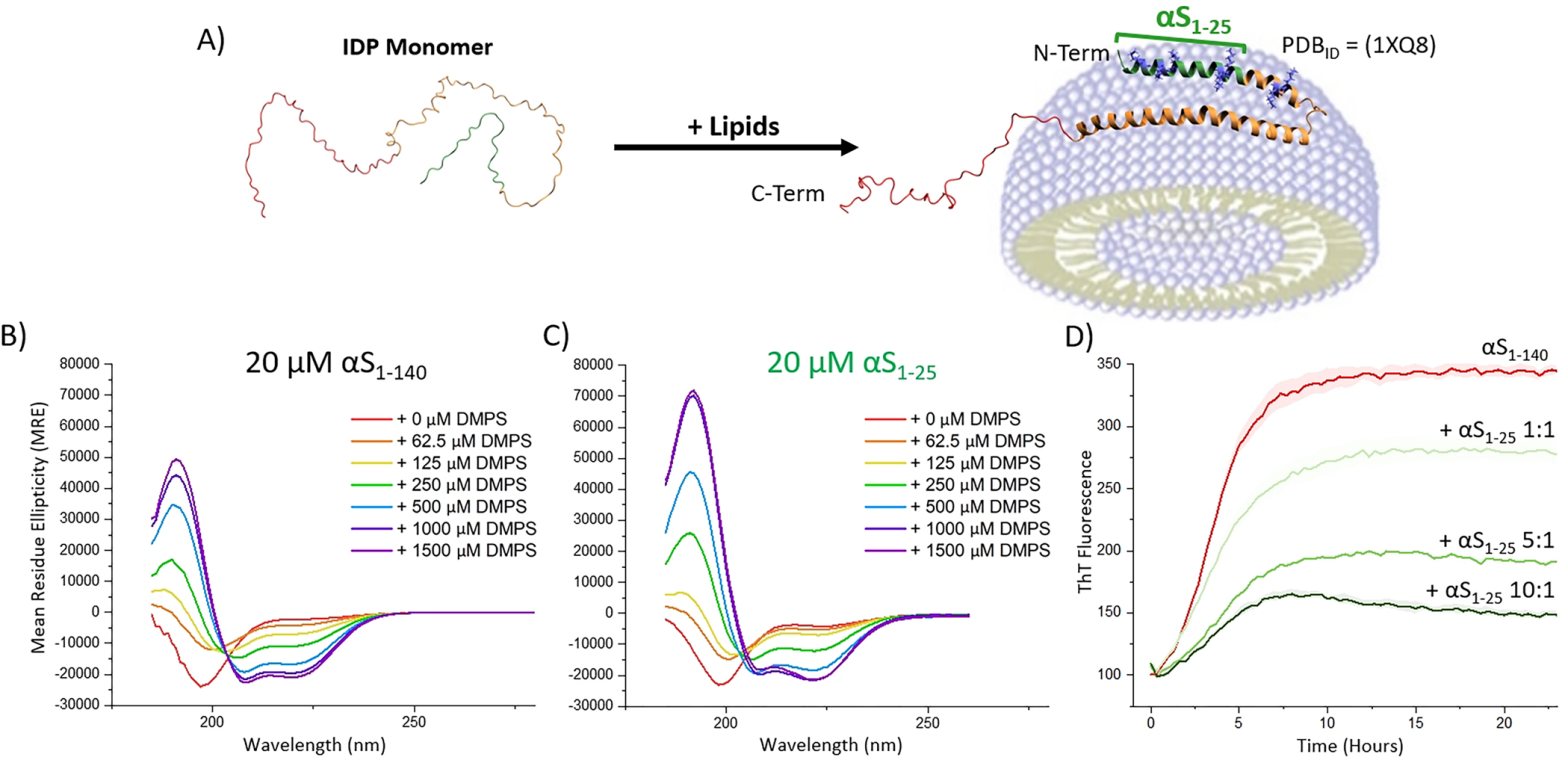
N-terminal fragments of αS_1–140_ retain lipid binding and lipid-induced aggregation inhibition properties. Adapted from Meade et al.^1^ (A) αS_1–140_ is an IDP in solution but adopts an α-helical conformation upon lipid binding (PDB_ID_ = 1XQ8 ^2–4^ ) (B) Circular dichroism confirms α-helicity of αS_1–140_ in the presence of DMPS SUVs. (C) A truncated N-terminal fragment of αS_1–25_ (82% of αS_1–140_ deleted) retains comparable lipid-induced helicity. (D) ThT aggregation assay shows that αS_1–25_ inhibits lipid induced aggregation of αS_1–140_ in the presence of DMPS SUVs.

## Lipid Binding Studies of Truncated αS_1–25_ Variants

A total of 14 constructs were designed to identify the shortest sequence capable of undergoing lipid-induced α-helix formation (Figure 2). During this iterative truncation process, we observed that even modest N-terminal deletions compromised lipid-induced helix-induction, while up to thirteen C-terminal residues could be removed without loss of function. This resulted in the removal of 92% of the αS parental sequence while still retaining lipid binding and α-helicity (fractional helicity (fH) = αS_1–25_ 58.1% vs αS_2–12_ 26.2%). As expected, the resulting 11mer (αS_2–12_) displayed a random coil conformation in the absence of lipid but underwent a coil-to-helix transition upon addition of DMPS small unilamellar vesicles (SUVs) (fH = 8.1 vs 26.2%) (Figure 2G). In its helical state, αS_2–12_ spans approximately three helical turns and features a positively charged N-terminus and negatively charged C-terminus, which may serve to offset the helical macrodipole and contribute to structural stabilization.^40^

**Figure 2:**
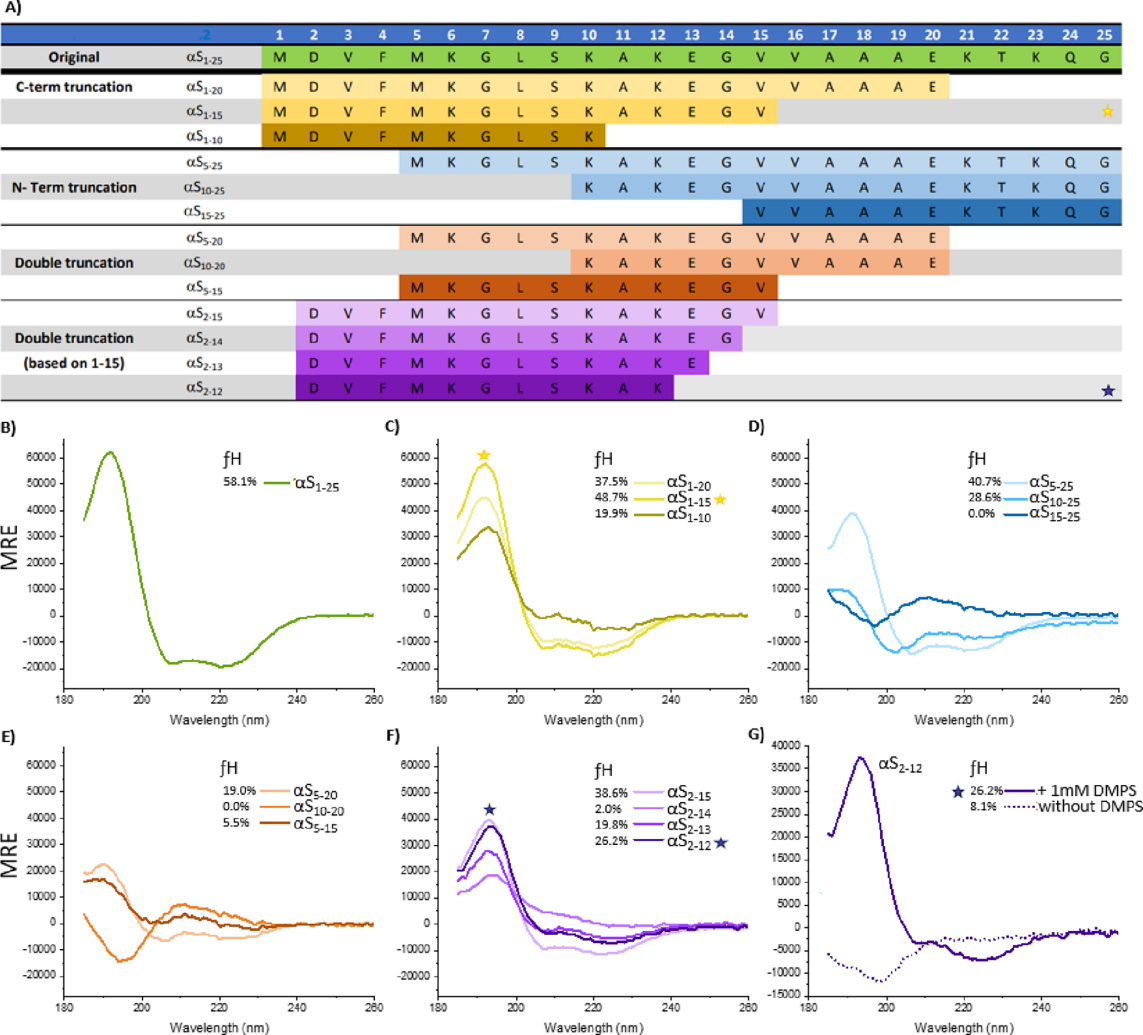
Lipid binding properties of αS_1–25_ truncations assessed by circular dichroism (A) Table summarizing N- and C- terminal truncations of the parent αS_1–25_ peptide. (B) CD spectra showing lipid binding of 20 μM αS_1–25_ in the presence of 1000 μM DMPS vesicles (C) C-terminal truncations (removal of up to 10 residues) do not significantly disrupt lipid binding, as assessed by CD. (D) N-terminal truncations of αS_1–25_. (E) lipid binding of N- and C-terminal truncations of αS_1–25_ (F) Further truncation of αS_1–15_ to αS_2–12_ results in a peptide that retains lipid-binding capacity. (G) Peptide αS_2–12_ adopts a random coil structure in aqueous buffer (dotted line; 8.1% fractional helicity), which shifts to a more helical conformation (solid line; 26.2% fractional helicity) upon interaction with DMPS SUVs. All CD spectra represent the mean of three independent measurements.

## Lactamization Induces Helicity of αS_2–12_ in the Absence of Lipid Vesicles

To overcome limitations related to peptide structural stability and biostability, one widely used strategy is to introduce backbone constraints that impose conformational stability and enhance binding affinity. Among these, lactam bridges, which are formed by cyclizing the peptide side chains to create isopeptide bonds, are particularly effective. Lactam constraints lead to entropic preorganization to impart enhanced peptide rigidity, reducing conformational flexibility, and enhancing resistance to proteolysis.^41,42^ In this study, we incorporated lactam constraints into the αS_2–12_ sequence to compensate for the loss of lipid-mediated helix induction, thereby improving both peptide stability and αS binding (Figure 3). Specifically, we employed i → i + 4 KD linkages (e.g., K1 → D5) to covalently pin one turn of the α-helix. This constraint type has been extensively used in peptide design by our group and others,^43–46^ and is favored over alternatives (e.g., DK, KE, EK, OD, DO, EO, OE; O = Orn), which, although chemically feasible, are less effective at inducing helicity in model systems.^43,47^

**Figure 3:**
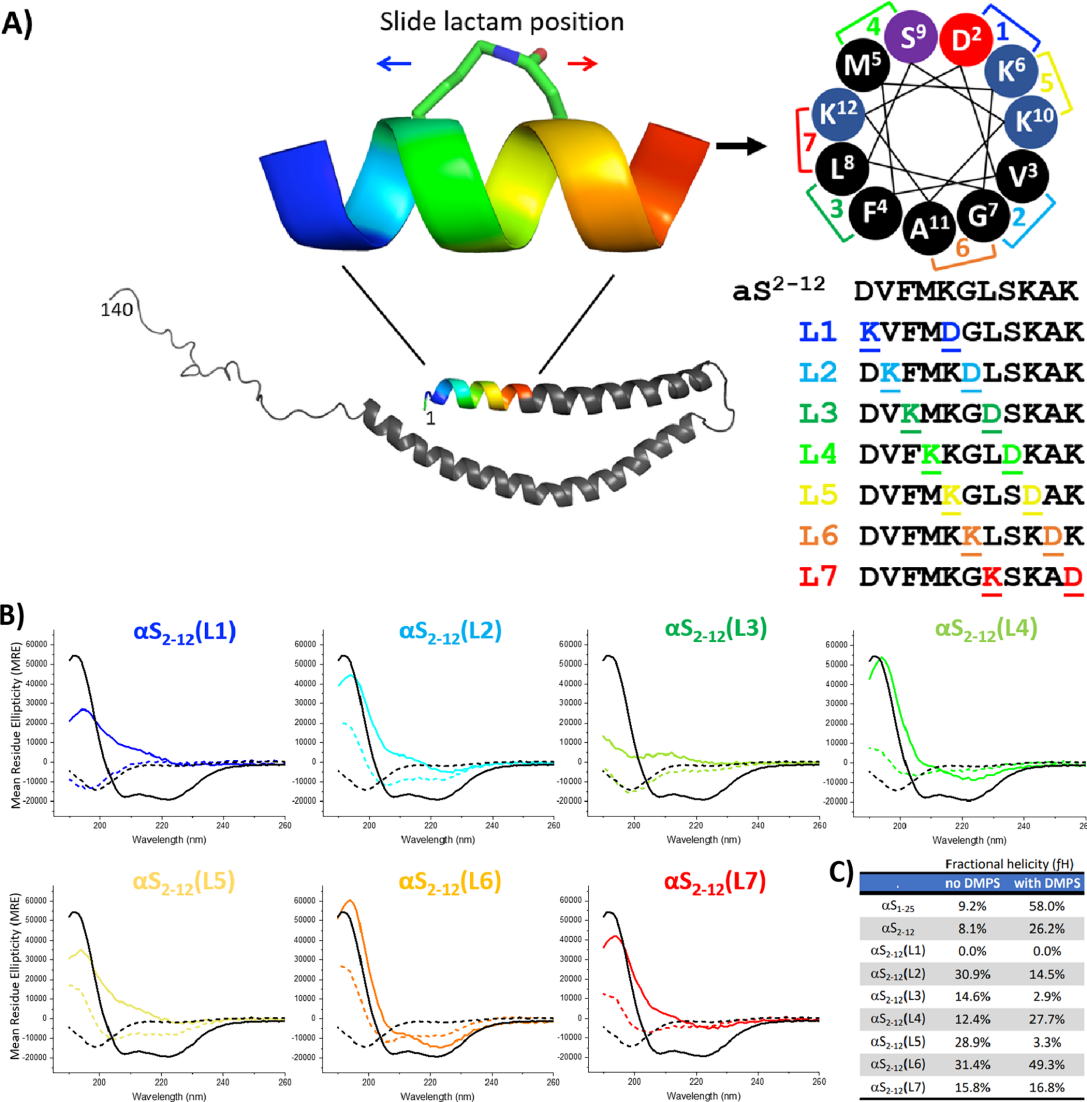
Lipid binding and helicity of αS_2–12_ lactams analogues assessed by circular dichroism (A) Seven lactam-constrained variants of αS_2–12_ were synthesized, each featuring a single (i → i + 4) side-chain linkage at a different helical turn position (B) CD spectra of each peptide (20 μM; color gradient blue to red) were recorded in the absence (dotted lines) and presence (solid lines) of 1000 μM DMPS vesicles. Full-length αS_1–25_ (black trace) is included for reference. (C) Table summarizing fractional helicity of each peptide with and without DMPS SUVs. Among the lactams, αS_2–12_ (L6) exhibited the highest helicity in both the absence (31.4%) and presence (49.3%) of lipid vesicles, representing a 23% increase in helicity relative to the unconstrained αS_2–12_ linear peptide. All spectra represent the mean of three independent measurements.

A K → D lactam scan was performed across all seven possible positions in the αS_2–12_ sequence, generating peptides αS_2–12_(L1-L7). All peptide constructs, both linear and lactamized, were synthesized via solid-phase peptide synthesis and analyzed for secondary structure using circular dichroism (Figure 3B). CD spectra were collected in the presence (solid line) and absence (dotted line) of DMPS SUVs to determine the individual and combined effects of (i) lactam bridging, (ii) lipid-induced folding, and (iii) synergistic effect upon α-helicity (Figure 3C). In the absence of the lipids, lactam bridging alone increased helicity in αS_2–12_(L2), αS_2–12_(L4), and, most notably, αS_2–12_(L6), which achieved a fractional helicity (fH) of 31.4%. These results demonstrate that a helix-promoting constraint can substitute for lipidic induction, with αS_2–12_(L6) representing the most effective construct for stabilizing α-helicity without membrane interaction.

## Lipid-Induced Aggregation Assays Determine the Effect of Lactam Position on αS_1–140_ Aggregation

The inhibitory effect of lactamized peptides, αS_2–12_L1-L7, on αS aggregation kinetics was evaluated using Thioflavin T (ThT) fluorescence (Figure 4), which quantifies amyloid fibril formation and its inhibition.^48^ Peptides were added at equimolar and 5-fold molar excess to 100 μM monomeric αS in the presence of 50 μM ThT, 50 μM DMPS, in 20 mM phosphate buffer (pH 6.5) at 30 °C. This data was used in conjunction with initial predictions of α-helical amphipathicity to assess how the K → D lactam position influences the ability of each peptide to inhibit lipid-induced aggregation. The most effective inhibition occurred when the lactam bridge was positioned on the hydrophobic face of the helical wheel, specifically in αS_2–12_(L2, L3, and L6). In contrast, placing the lactam on the polar, solvent-exposed face (αS_2–12_(L1, L4, and L7)) enhanced αS aggregation, likely due to the loss of key polar or charged residues required for inhibitory activity. These findings suggest that strategic placement of the lactam constraint is critical both to preserve helix-stabilizing features and to maintain interactions essential for blocking lipid-induced nucleation of αS.

**Figure 4:**
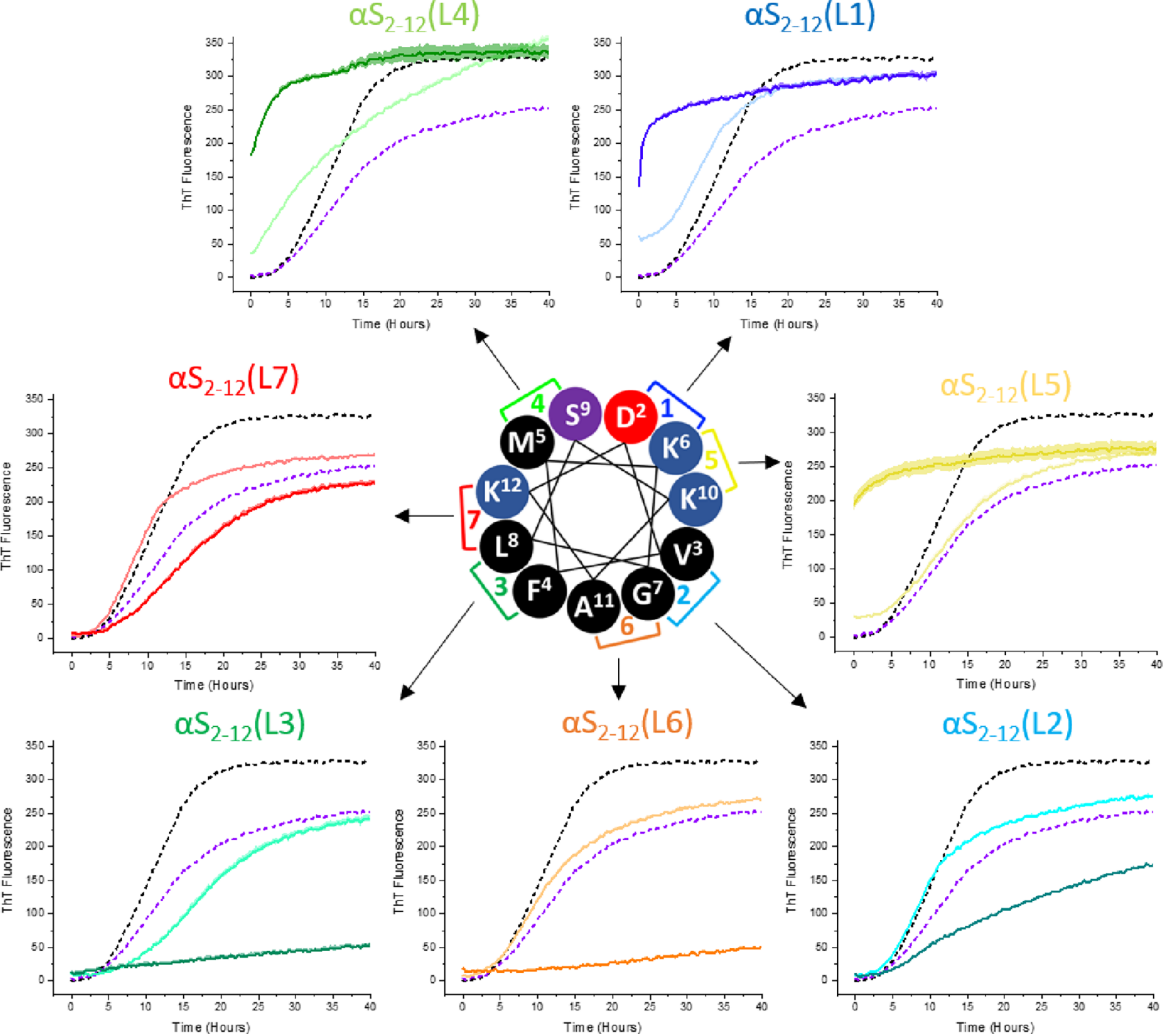
Lactam scan identifies optimal constraint positions for inhibiting αS_1–140_ aggregation (A) ThT kinetic aggregation assays were performed in 20 mM phosphate buffer (pH 6.5) at 30 °C, using 100 μM αS_1–140_, 50 μM ThT, and 50 μM DMPS SUVs. αS_2–12_ lactam analogues (L1-L7) were tested as inhibitors at two concentrations: 100 μM (light traces) and 500 μM (dark traces). Aggregation of αS_1–140_ alone is shown as a black dashed line; linear, unconstrained αS_2–12_ at 500 μM is shown as a purple dashed line. Lactams αS_2–12_(L6) and αS_2–12_(L3) demonstrated the greatest inhibition of αS_1–140_ aggregation. In contrast, lactams bridging the hydrophilic face of the helix (L1, L4, L7)) appear to enhance aggregation. Peptides constrained on the hydrophobic face (L2, L3 and L6) were the most effective at suppressing DMPS-induced aggregation, highlighting the importance of helix face orientation in modulating activity. Data shown as mean ± standard error (*n* = 3).

## αS_2–12_(L6) Is a Potent Dose-Dependent Inhibitor of Lipid-Induced αS_1–140_ Aggregation

Given the differential effect of the helix-constrained peptides on lipid-induced αS aggregation, lactam bridge L6 emerged as the most effective construct for enhancing both peptide helicity and inhibitory activity. To further evaluate its potency, αS_2–12_(L6) was tested in a dose–response assay across a stoichiometric range from 0.25:1 to 10:1, using 100 μM monomeric αS in the presence of 50 μM ThT and 50 μM DMPS (20 mM phosphate buffer, pH 6.5, 30 °C). This revealed dose-dependent inhibition, culminating in near-complete inhibition of αS aggregation at a 10:1 ratio (Figure 5A).

**Figure 5:**
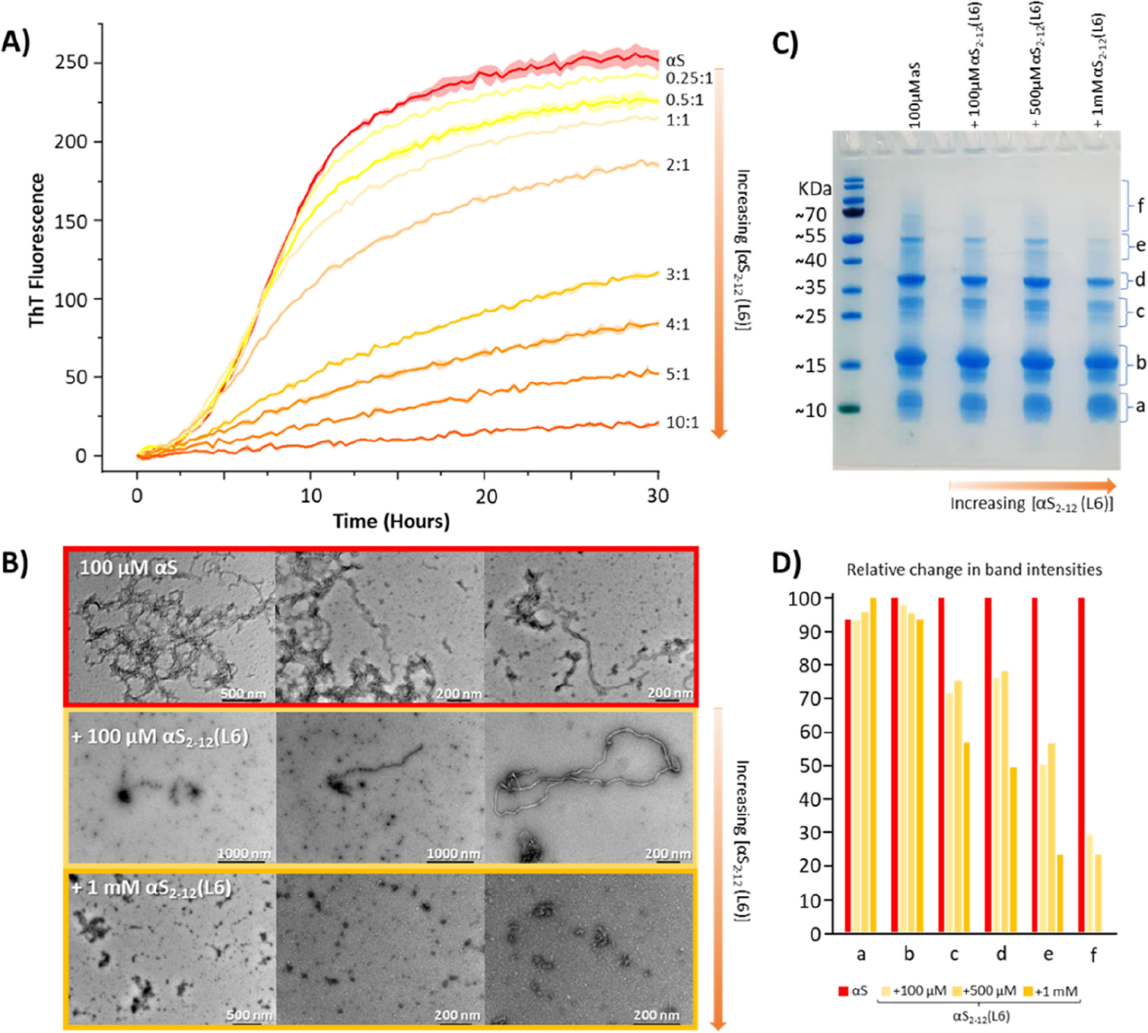
αS_2–12_(L6) is a potent, dose-dependent inhibitor of αS_1–140_ aggregation (A) ThT kinetic assays were performed with 100 μM αS_1–140_, 50 μM ThT and 50 μM DMPS in 20 mM phosphate buffer (pH 6.5) at 30 °C, in the presence of increasing concentrations (0–100 μM) of αS_2–12_(L6). Aggregation is progressively suppressed with increasing inhibitor concentration, with complete inhibition observed at 100 μM. Data shown as mean ± standard error (*n* = 3). (B) TEM images from the same end point ThT assays reveal fibril structures in the absence of αS_2–12_(L6) (red) and substantial suppression of fibril formation in its presence (orange). (C) SDS-PAGE of protein samples collected at the ThT end point, following PICUP, shows a reduction in oligomeric αS_1–140_ species with increasing concentrations of αS_2–12_(L6). (D) Densitometric analysis of SDS-PAGE band intensities using ImageJ confirms a dose-dependent decrease in higher-order oligomer bands (bands c–f), correlating with increasing αS_2–12_(L6) concentration.

To probe aggregate formation, end point samples (30 h) were analyzed using photoinduced cross-linking of unmodified peptides (PICUP)^49^ and visualized by SDS-PAGE (Figure 5C). Increasing concentrations of αS_2–12_(L6) corresponded with reduced intensity of low molecular weight aggregate bands (c–f) and preservation of distinct monomer bands (a, b). These may represent different αS conformers with band b, possibly corresponding to the disordered monomer, and band a to the helical membrane-bound form. Notably, the most rapidly migrating monomer band slightly increased with αS_2–12_(L6) concentration, potentially indicating a peptide-bound aggregation-resistant conformation.

Transmission electron microscopy (TEM) of end point samples supported these findings (Figure 5B). In the absence of αS_2–12_(L6), densely packed fibrils were observed. At a 1:1 ratio, fibril formation was substantially reduced, while at a 10:1 ratio, amyloid fibrils were completely absent, confirming the peptides’ ability to inhibit lipid-induced αS aggregation. Additional lipid-induced aggregation end point TEM data is provided within Supplementary Figure 7.

## αS_2–12_(L6) Has No Effect on αS_1–140_ Aggregation by Agitation

As previously reported for αS_1–25,_ ^1^ kinetic analysis of ThT binding studies performed by agitating αS_1–140_ with a glass bead (i.e., in the absence of lipids) showed no inhibition of aggregation of αS_1–140_ by αS_2–12_(L6). The kinetics of agitation-induced aggregation of 100 μM αS_1–140_ were followed, with increasing concentrations of αS_2–12_(L6) (0–1000 μM), using a single 3 mm glass bead to agitate the solution (Figure 6A). TEM analysis of the end point aggregates (Figure 6B) presented long straight fibrils with a twisted structure, like those previously described in the literature.^35^ This suggests that αS_2–12_(L6) only inhibits αS_1–140_ aggregation in the presence of lipids, and does not bind to or inhibit αS_1–140_ when it is in solution.

**Figure 6:**
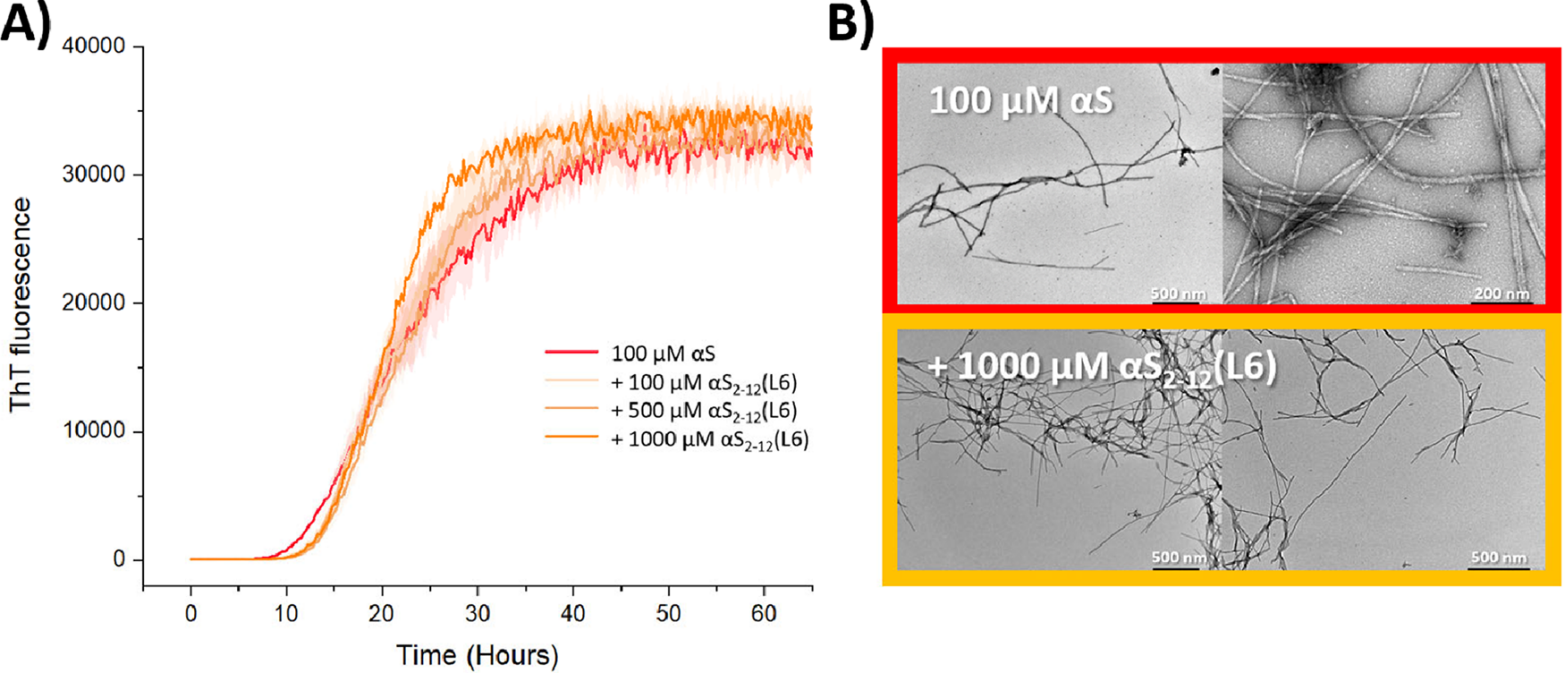
αS_2–12_(L6) has no effect on the agitation induced aggregation of αS_1–140_. (A) ThT kinetics of 100 μM αS_1–140_ and a single 3 mm glass bead to agitate the solution presented no change in aggregation with increasing concentrations of αS_2–12_(L6) (0–1000 μM). (B) Aggregates formed presented straight fibrils, consistent with those previously reported with αS_1–140_ aggregation. Data shown as mean ± standard error (*n* = 3).

## Structural Characterization of αS_2–12_(L6) in 50% TFE Using NMR

To further characterize the structural impact of the lactam constraint in αS_2–12_(L6), 2D NMR spectra were acquired to determine the solution structure. To mimic the lipidic membrane environment, spectra were collected in 50% TFE, a solvent known to stabilize the helical conformation and previously used for membrane-mimetic structural studies.^1,50,51^ The NMR solution structure revealed a high degree of α-helicity across the 20 lowest energy conformers (Figure 7). These structures were highly convergent, with a backbone RMSD of 0.43Å and no violations of distance or dihedral restraints (Supplementary Table 1). As expected, the K6 → D10 lactam constraint stabilized the C-terminal region, reducing the degree of C-terminal fraying (Figure 7B). The peptide maintained its amphipathic character, with the hydrophobic lactam forming on the hydrophobic face of the helix (Figure 7C), consistent with its membrane-binding and aggregation-inhibitory function.

**Figure 7:**
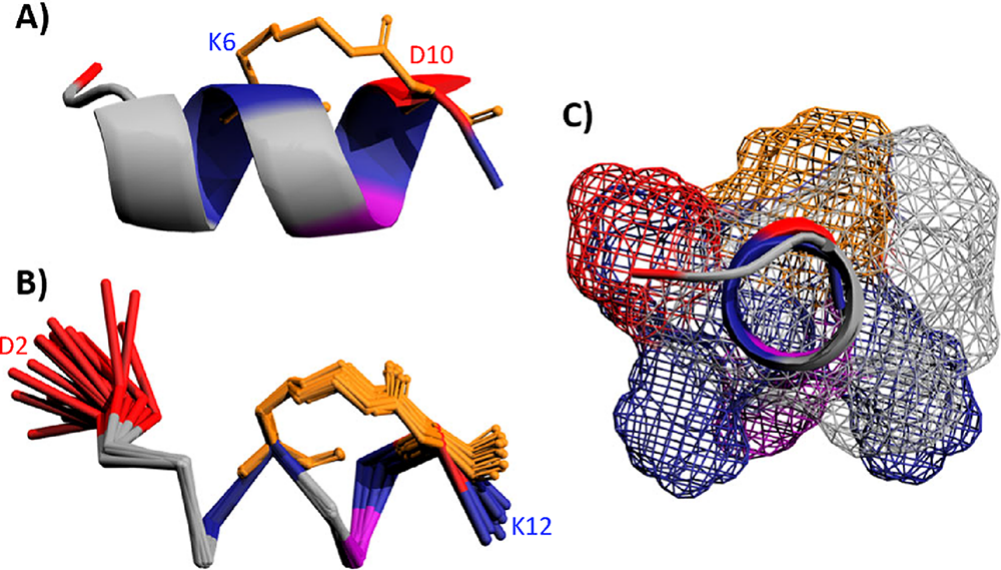
Solution NMR structure of αS_2–12_(L6). (A) Lowest-energy structure of αS_2–12_(L6). (PDB 8OL8), showing an amphipathic α helix with side chains colored by chemical character: positively charged (blue), negatively charged (red), polar (purple), and the lactam constraint (orange). (B) Ribbon ensemble of the 20 lowest energy conformers generated from the final structure calculation, demonstrating high structural convergence. (C) Solvent-accessible surface representation of αS_2–12_(L6), viewed along the helical axis from the N-terminus, illustrates the distribution of exposed side chains and the amphipathic nature of the helix. The helical form comprises approximately three turns, with each position in the helical wheel occupied. The presence of an N-terminal Asp (D) and a C-terminal Lys (K), may further stabilize the peptide.

## NMR Reveals That αS_2–12_(L6) Inhibits Lipid-Induced Aggregation by Preserving Native Monomeric αS_1–140_

Preservation of monomeric αS under lipid-induced nucleation conditions by αS_2–12_(L6) was confirmed at atomic resolution by using ^1^H–^15^N heteronuclear single quantum coherence (HSQC) NMR spectroscopy. Uniformly ^15^N-labeled αS (100 μM) was incubated with 50 μM DMPS in 20 mM sodium phosphate buffer (pH 6.5) either with or without a 1:1 ratio of αS_2–12_(L6). HSQC spectra were recorded at 30 °C on day 0 for both samples (Figure 8A,B). After 6 days at room temperature, the sample lacking αS_2–12_(L6) developed visible, pelletable aggregates, while the peptide-treated sample remained clear. Aggregated material was removed via centrifugation on day 6 (16,000 rpm, 30 min), and HSQC spectra were acquired from the supernatants (Figure 8C,D). The persistence of well-dispersed backbone amide peaks in the peptide-treated sample confirms that αS_2–12_(L6) maintains αS_1–140_ in its monomeric state, preventing lipid-induced aggregation.

**Figure 8:**
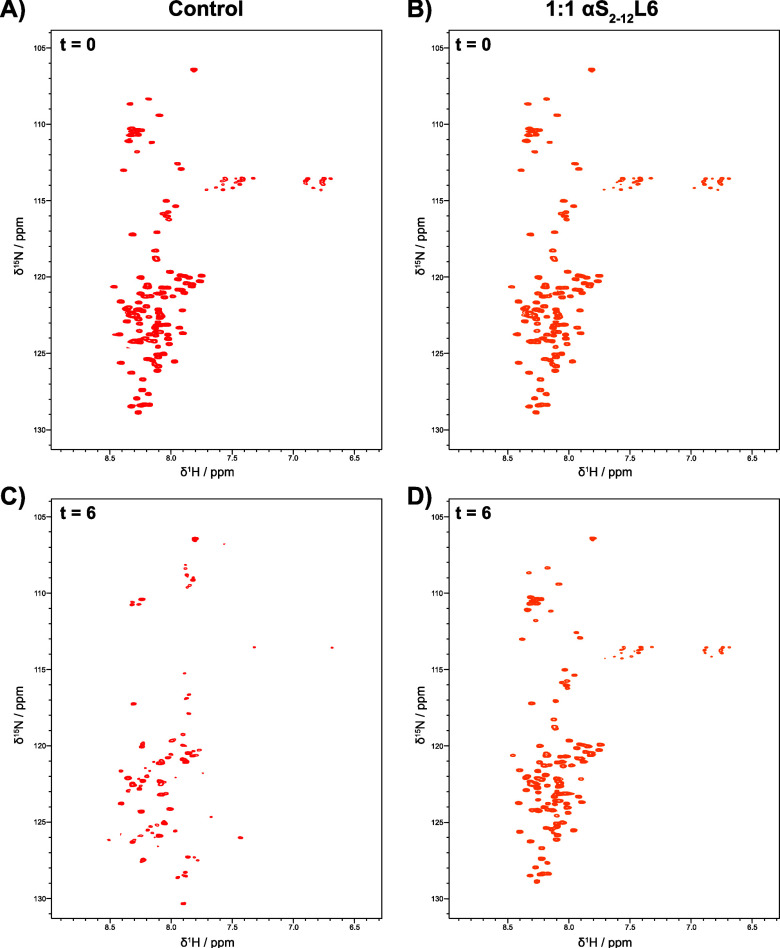
^1^H–^15^N HSQC spectra of αS_1–140_ in the presence of αS_2–12_(L6) over time. HSQC spectra were acquired for 100 μM 15N-labeled αS_1–140_ with 50 μM DMPS in 20 mM sodium phosphate buffer (pH 6.5) at 30 °C (A) Time (*t*) = day 0 spectrum of αS_1–140_ alone, showing well-resolved cross-peaks consistent with a disordered monomer. (B) Spectrum of αS_1–140_ at day 0 in the presence of αS_2–12_L6 at a 1:1 molar ratio, showing spectral features comparable to those of panel A. (C) Spectrum of αS_1–140_ alone after 6 days of incubation. Aggregated material was removed by centrifugation prior to acquisition; the remaining soluble protein displays substantial peak loss and broadening, consistent with aggregation. (D) Spectrum of αS_1–140_ with αS_2–12_(L6) after 6 days of incubation. The preservation of cross-peaks similar to those observed at day 0 indicates that αS_2–12_(L6) protects monomeric αS_1–140_ from aggregation or degradation under these conditions.

HSQC spectra confirmed that in the absence of αS_2–12_(L6), and despite the removal of visible aggregates, the resulting ^1^H–^15^N HSQC spectra showed a dramatic reduction in spectral quality. Most amide resonances were lost, and several new resonances were observed, consistent with the formation of oligomeric or intermediate species. In contrast, the sample supplemented with αS_2–12_(L6) retained a high-quality ^1^H–^15^N HSQC spectrum after 6 days, with all expected 133 backbone amide cross peaks present (excluding the five prolines and residues 1 and 2), and signal dispersion was comparable to both day 0 controls. A plot of relative peak intensities (Supplementary Figure 4) displayed values close to 1.0 across residues 20–140, supporting the conclusion that monomeric αS was preserved in the presence of αS_2–12_(L6). In the absence of the peptide, significant peak broadening, signal loss, and emergence of new peaks might suggest the presence of low molecular weight aggregates that retain some interesting, NMR observable, flexible regions^52^ which are consistent with TEM data (Figure 5B, Supplementary Figure 7). Given that both samples were derived from the same purified protein batch, sample degradation due to sample-related issues such as proteolysis is unlikely, as the αS_2–12_(L6)-treated sample remained fully intact. Together, these data indicate that αS_2–12_(L6) inhibits lipid-induced aggregation of αS, thereby preventing the formation of higher-order oligomers and downstream aggregation. Finally, we confirmed that the lactam-constrained peptide αS_2–12_(L6) partially displaces α-synuclein from DMPS vesicles, as shown by increased ^1^H–^15^N HSQC peak intensities across the N-terminal and NAC regions (Supplementary Figure 5). This effect was not observed with the linear control peptide, supporting a mechanism where the constraint specifically disrupts lipid-induced nucleation rather than general lipid binding (see SI).

## Lactamized αS_2–12_(L6) Provides Increased Serum Stability Relative to Linear αS_2–12_

The impact of the lactam constraint in αS_2–12_(L6) on peptide stability was assessed in human serum, using the native linear sequence αS_2–12_ as a control (Figure 9). After only 5 h, the linear αS_2–12_ had degraded by 96%, whereas αS_2–12_(L6) showed only 14% degradation. Extended incubation up to 48 h revealed that 16% of the constrained peptide remained intact, demonstrating the enhanced protease stability conferred by the lactam bridge.

**Figure 9:**
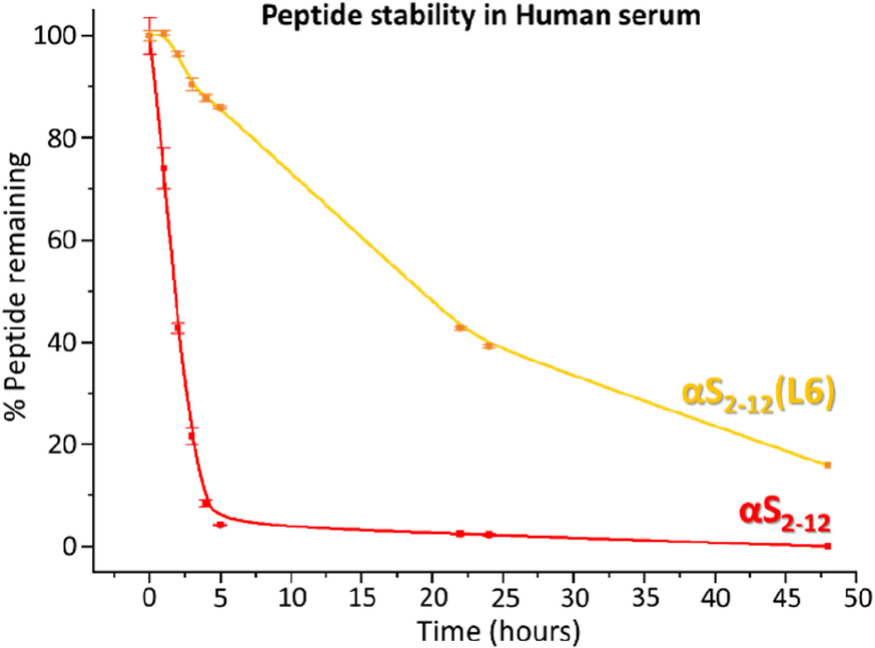
Lactamization of αS_2–12_(L6) enhances peptide stability in human serum. Serum stability was assessed by incubating αS_2–12_ (linear) and αS_2–12_(L6) (lactamized) in human serum at 37 °C. Peptide concentration was quantified by analytical HPLC at selected time points and normalized to *t* = 0. After 5 h, 96% of the linear αS_2–12_ was degraded, whereas only 14% of the lactamized αS_2–12_(L6) had been lost, indicating substantially increased protease resistance conferred by the lactam constraint. Data represent the mean of three independent experiments; error bars indicate the standard error of the mean.

## αS_2–12_(L6) Is Nontoxic to Cell Culture and Demonstrates Robust Uptake in SH-SY5Y Cells

Peptides labeled with Rhodamine-B (RhoB) were readily taken up by SH-SY5Y neuroblastoma cells, as confirmed by live fluorescence microscopy and quantification of RhoB-labeled puncta (Figure 10). SH-SY5Y cells were incubated with RhoB-labeled peptides and live imaged after ∼36 h, revealing dose-dependent intracellular accumulation of αS_2–12_(L6) and its linear controls. Punctate staining was localized primarily to the perinuclear region (Figure 10A), with both the abundance and intensity of rhodamine signal increasing with peptide concentration (Figure 10B). No differences in uptake were observed among the three peptide variants. Importantly, treatment with 10 or 20 μM peptide was nontoxic, as determined by Alamar Blue (Figure 10C) and ToxiLight (Figure 10D) assays, which showed no significant cytolysis or impairment of mitochondrial function relative to baseline. This assay clearly demonstrates that the lipid-binding amphipathic nature of the peptides enables efficient cell membrane penetration without the need for additional cell-penetrating appendages. This effect is further amplified by the stabilizing effect of the lactam constraint.

**Figure 10:**
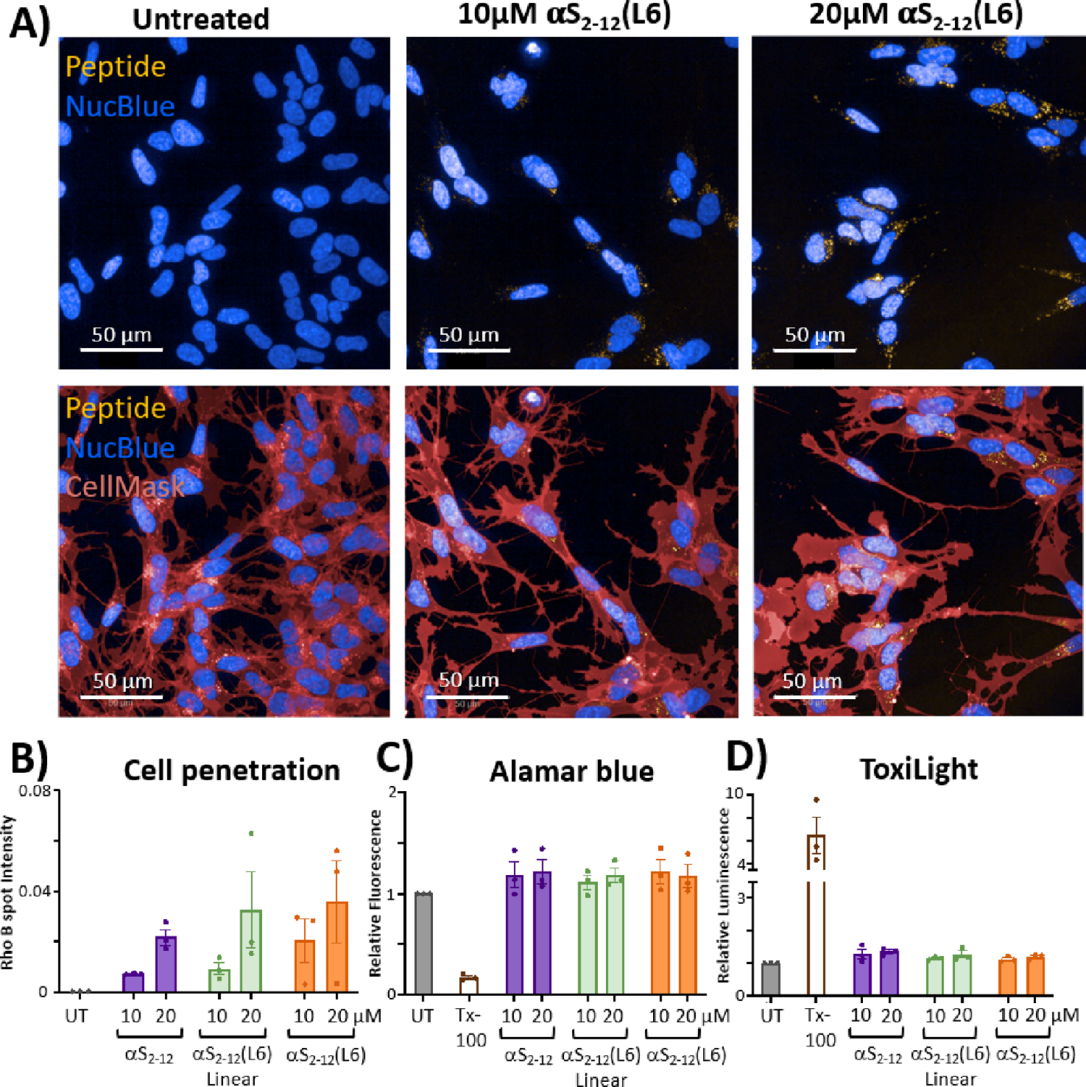
Peptide uptake and cytocompatibility in SH-SY5Y cells. (A) Representative fluorescence microscopy images of SH-SY5Y cells treated with peptide L6 (10 and 20 μM) for 33 h, showing intracellular peptide uptake. Untreated cells are shown for comparison. Nuclei are stained with NucBlue (blue), cytoplasm with CellMask (red), and peptides are visualized in yellow (scale bar = 50 μm). Images shown are z-projections spanning 8 μm, capturing all in-focus fluorescence signals across the stack. (B) Quantification of intracellular uptake, represented as the number of RhoB-labeled peptide puncta relative to local background signal at each location, indicating peptide internalization. (C) Cytotoxicity of peptides αS_2–12_, αS_2–12_(L6) (constrained), and αS_2–12_L6 (linear) assessed by Alamar Blue and ToxiLight assays in SH-SY5Y cells. Viability is expressed relative to that of untreated controls and Triton X-100 (Tx-100) as a positive control. No significant toxicity was observed across the 0–20 μM concentration range. Each data point represents the mean of at least three technical replicates, each from independent experimental repeats involving separate platings of the SH-SY5Y cells. Note: The SH-SY5Y cells used in this study expressed low endogenous levels of αS.

## αS_2–12_(L6) Rescues a *C. elegans* Model of an αS_1–140_-Induced Movement Disorder

To evaluate whether the effects of αS_2–12_(L6) observed in biochemical and cellular assays are recapitulated in a complex whole organism, we used the NL5901 *C. elegans* model, in which αS:YFP is overexpressed under the unc-54 promoter in muscle tissue.^53^ This widely adopted model recapitulates αS-induced movement disorders and has been extensively used to study inclusion formation and locomotor defects.^54–59^ Overexpression of αS leads to age-dependent loss of motility and visible fluorescent inclusions in muscle cells (Figure 11B). This presents as a loss of mobility, which can be measured by determining the thrashing rate. At larval stage L4, the worms were exposed to a range of αS_2–12_(L6) concentrations (0–100 μM), spotted directly onto agar plates (Figure 11A). Following incubation, wild-type N2 controls, which do not express αS, showed no change in thrashing rate, indicating that αS_2–12_(L6) is nontoxic over the tested range. In contrast, NL5901 worms displayed a progressive loss of motility by day 5 of adulthood, which was rescued by αS_2–12_(L6) in a dose-dependent manner, with near-complete restoration at 100 μM (Figure 11C). αS_2–12_(L6) was readministered to the worms at day 7 of adulthood (Figure 11A) to maintain peptide exposure across developmental stages, as serum stability experiments suggested that it is degraded after a few days (Figure 9). By day 13 of adulthood, clear, distinct, YFP-labeled inclusions were observed in untreated NL5901 worms (Figure 11B), and these were substantially reduced in peptide-treated populations (Figure 11D,E). These findings demonstrate that αS_2–12_(L6) is bioavailable in vivo, crosses biological membranes, and effectively inhibits the aggregation of αS into toxic conformations in a complex multicellular environment.

**Figure 11:**
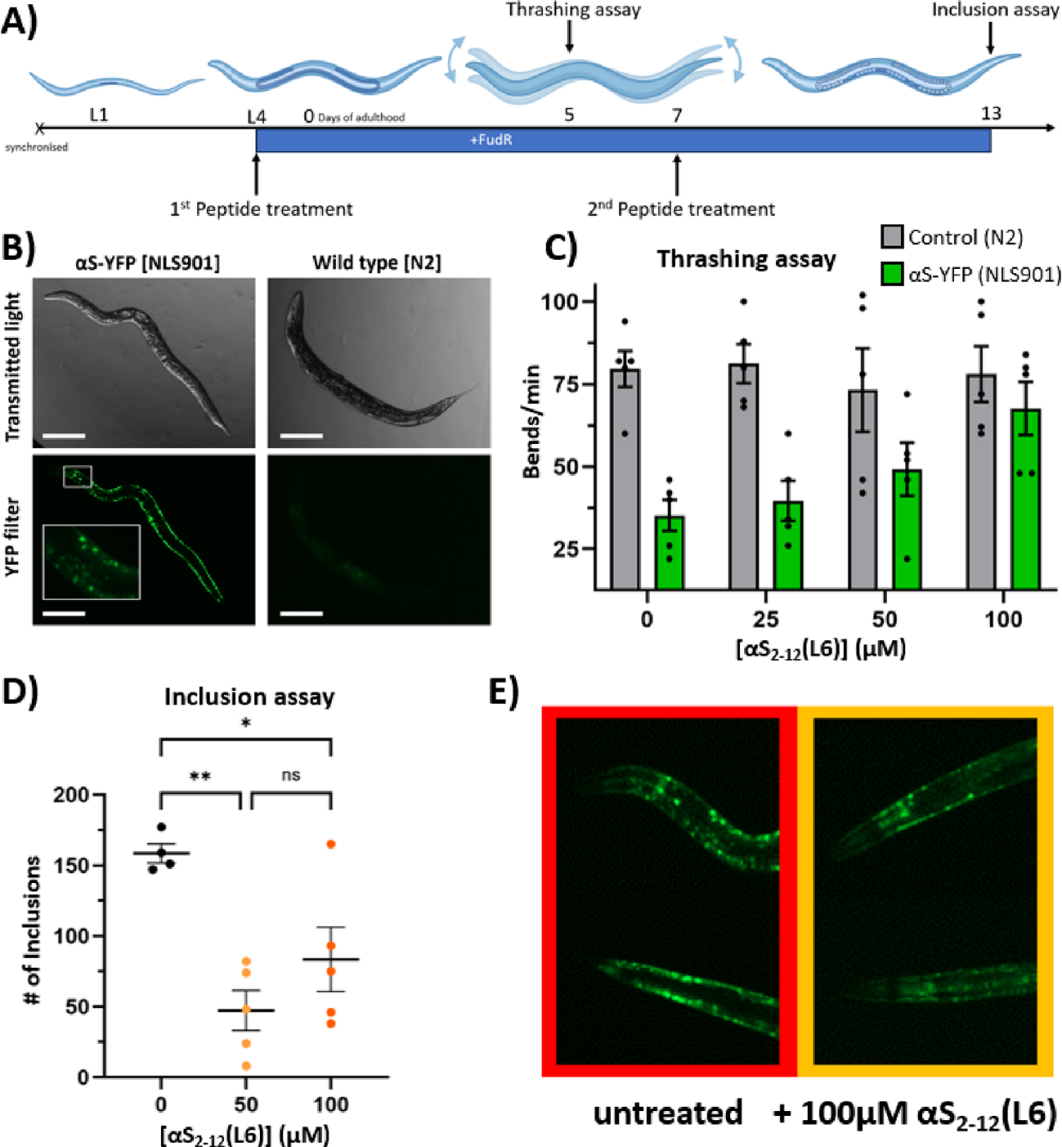
αS2–12­(L6) treatment rescues disease phenotype in *C. elegans* expressing α-synuclein–YFP. (A) NL5901 worms expressing α-synuclein–YFP (αS–YFP) in body wall muscle were treated with αS_2–12_(L6) at the L4 larval stage. Mobility was assessed by thrashing frequency on Day 5 (D5) of adulthood. Worms were redosed on adult day 7 (D7), and inclusion formation was assessed via fluorescence microscopy on adult day 13 (D13). (B) On D13, NL5901 worms displayed prominent αS–YFP fluorescent inclusions along the body wall in the absence of peptide, while control N2 worms showed no fluorescence (scale bar = 200 μm, 5 worms analyzed per condition). (C) Thrashing assays performed on D5 revealed that peptide treatment had no adverse effect on N2 wild-type worms. In contrast, NL5901 worms showed significantly reduced motility (*P* < 0.01) without treatment, which was rescued in a dose-dependent manner by αS_2–12_(L6). (D) Peptide treatment significantly reduced αS–YFP inclusion burden in NL5901 worms on D13 (*P* < 0.01) (5 worms analyzed per condition). (E) Representative images of worm heads showing αS–YFP fluorescence with and without peptide treatment. A reduction in the level of fluorescent inclusions is observed in peptide-treated worms. Full-body images are provided in Supplementary Figure 6.

## Conclusions

Previous studies have demonstrated that αS_1–25_ can inhibit αS aggregation in the presence of lipid vesicles^1^ (Figure 1D). To minimize the peptide while retaining function, we generated a series of N- and C-terminal αS_1–25_ truncations of αS_1–25_. From this, we identified αS_2–12_ as the shortest sequence capable of undergoing lipid-induced α-helix formation (Figure 2). Notably, even the removal of two N-terminal residues severely impaired helix-induction, while deletion of up to 13 C-terminal residues was tolerated, resulting in a 92% deletion of the full αS sequence. The resulting 11mer (αS_2–12_) adopted a mostly random coil in aqueous solution (8.1% helicity) without lipidic stabilizing agents, containing insufficient internal hydrogen bonds to assemble into an α-helix. However, in the presence of DMPS lipids, αS_2–12_ displayed 26.2% helicity (Figure 2E). Importantly, this helicity can be substituted for by the addition of a constraint, without the need for lipid (31.4% helicity without lipid (Figure 3B, orange line, constraint position 6). This modification constrains the αS_2–12_ sequence by covalently pinning one (i → i + 4) turn of the α-helix, entropically preorganizing the peptide, to compensate for the loss of lipid interaction to ultimately facilitate αS binding and peptide stability. Given that the αS_2–12_ region is critical for helix-induction and oligomerization within αS,^23–25^ the constrained variant αS_2–12_(L6) effectively blocks aggregation as demonstrated by ThT fluorescence, CD, and TEM. It also exhibits marked proteolytic stability, persisting over several days, relative to the linear αS_2–12_, which is fully degraded within hours. In SH-SY5Y cells, αS_2–12_(L6) was nontoxic and, owing to the cationic and amphipathic character, could readily enter cells without additional modifications (e.g., lipidation or cell-penetration sequences). Finally, in the NL5901 *C. elegans* model of αS overexpression, αS_2–12_(L6) was shown to enter the organism, rescue the thrashing motility phenotype, and significantly reduce protein inclusions to demonstrate in vivo bioactivity and therapeutic potential. The peptide-membrane-binding properties potentially contribute to bioavailability and function inside worms.

In summary, strategic downsizing of αS_1–25_ followed by incorporation of a lactam bridge at position 6 in the αS_2–12_ peptide has proven highly effective for enhancing structural stability, biostability, cellular uptake, and ultimately antiaggregatory activity. High-resolution NMR confirmed that the lactam constraint stabilizes a well-defined, amphipathic α-helical structure, further supporting its role in functional engagement with αS_1–140_. In addition, new HSQC displacement experiments (Supplementary Figure 5) show that the lactam-constrained peptide partially disrupts αS binding to DMPS vesicles, an effect not observed for the linear control. Complementary PICUP analysis (Figure 5C) reveals an altered αS oligomerization pattern in the presence of αS_2–12_(L6), consistent with additional effects on early self-association events beyond membrane competition. Moreover, solution-state NMR of lipid-induced aggregation reactions (Figure 8) shows that αS_2–12_(L6) increases the proportion of soluble αS species relative to controls, suggesting inhibition of early aggregate formation. This helix-inducing constraint approach not only protects the peptide from degradation but also promotes membrane penetration,^60–63^ aligning with broader advances in peptide design that enable potent modulation of protein function. Together, these findings support a mechanism in which αS_2–12_(L6) both competes for membrane binding and perturbs pathogenic αS self-assembly. This approach offers timely and promising potential for the development of next-generation peptide-based therapeutics.^64,65^

## Methods

### Protein Expression and Purification of Human Wild-Type αS

Wild-type human αS was recombinantly expressed and purified using a method adapted from previously published protocols^66,67^ (see Supplementary Figure 1). Briefly, the pET21a plasmid encoding human wild-type αS_1–140_ (Addgene; deposited by the Michael J. Fox Foundation) was transformed into *E. coli* expression cell line BL21 (DE3) cells. A 10 mL overnight culture in 2xYT medium with 100 mg L^–1^ ampicillin was used to inoculate 1 L 2xYT cultures (also containing 100 mg L^–1^ ampicillin). Cultures were grown at 37 °C with shaking (200 rpm) to OD_600_ = 0.6–0.8, then induced with 1 mM isopropyl-1-thio-D-galactopyranoside (IPTG) for 4 h under the same conditions (Innova 44 Incubator shaker, New Brunswick Scientific). Cells were harvested by centrifugation (4600*g)*, resuspended in 40 mL of 20 mM Tris buffer (pH 8) containing one Complete protease inhibitor tablet (Roche), and freeze–thawed at −20 °C before lysis by sonication. Cell debris was removed by centrifugation (48,400*g)*, and the supernatant was collected and boiled at 95 °C for 10 min. Precipitated proteins were removed by centrifugation (18,500*g)*. Ammonium sulfate was added to the supernatant to 30% saturation (0.176 g mL^–1^), and the solution was left shaking at RT for 1 h. The resulting precipitate, enriched in αS, was harvested by centrifugation (18,500*g)* and resuspended in 50 mL of 20 mM Tris buffer (pH 8) by gentle agitation at 4 °C. The protein was purified by anion exchange chromatography using a 5 mL hiTRAP Q HP column (GE Healthcare) on an ÄKTA pure purification system (GE Healthcare) to remove nucleic acid and protein contaminants. Pooled fractions were further purified and buffer-exchanged by size exclusion chromatography (SEC), using a HiLoad 16/60 Superdex 75 pg (GE Healthcare) prepacked purification column, in either 20 mM sodium phosphate buffer (pH 6.5). Only monomeric αS fractions from the peak apex were collected. The concentration of the purified αS was determined by the absorbance at 280 nm (ε = 4836 M^–1^ cm^–1^) using a 2 mm quartz cuvette. Purified αS was used on the same day as SEC.

Purity of the αS following SEC was confirmed by SDS-PAGE, and the correct molecular weight was confirmed by mass spectrometry. Mass analysis was performed using a Dionex Acclaim RSLC Polar Advantage II (PA2) column (2.2 μm, 120 Å, 2.1 mm × 50 mm; Thermo Fisher Scientific, California, USA). The deconvoluted average mass was determined to be 14460.16 Da, consistent with the expected mass of wild-type Human αS.

### Production and Purification of Peptides

Peptides were synthesized on a 0.1 mmol scale using a Liberty Blue microwave peptide synthesizer (CEM) with Rink amide ChemMatrix resin (PCAS BioMatrix) and standard Fmoc solid-phase chemistry. Each amino acid was introduced through repeated cycles of coupling, deprotection, and washing. Coupling was carried out using benzotriazol-1-yloxytripyrrolidinophosphonium hexafluorophosphate (PyBOP, 0.5 M) and diisopropylethylamine (DIPEA, 1M) in DMF, while deprotection was performed in 20% piperidine in DMF containing 5% formic acid to prevent aspartamide formation. Fmoc-Lys­(Mtt)–OH (Fluorochem) and fmoc-Asp­(O-2-PhiPr)–OH (Merck) were employed at the relevant positions to enable orthogonal side chain deprotection and lactamization.

For unlabeled peptides, the peptide N-terminus was acetylated by a final reaction with 20% (v/v) acetic anhydride, DIPEA (1M, 4 mL) in DMF for one h at room temperature. For RhoB-labeled peptides, RhoB was conjugated to the N-terminus by double coupling with RhoB (5 equiv) (Merck), DIPEA (10 equiv), and PyBOP (5 equiv) for a total time across both couplings of 24 h at 50 °C.

Orthogonal side chain deprotection of Lys­(Mtt) and Asp­(O-2-PhiPr) was performed by washing (3×) the resin with dichloromethane (DCM), then incubating the resin in 2% trifluoroacetic acid (TFA) in DCM for 2 min (10×). The resin was then washed with DCM (3×) and then DMF (3×). The deprotected side chains were double coupled using diisopropylcarbodiimde (DIC) (5 equiv) and Oxyma Pure (2.5 equiv) in DMF at 50 °C for 3 h, then overnight.

Following synthesis, peptides were cleaved from the resin and globally deprotected by incubating in cleavage solution (95% TFA, 2.5% triisopropylsilane, 2.5% water) for 4 h at room temperature. The resin was removed by filtration, and the crude peptide precipitated using cold diethyl ether (−80 °C), then collected by centrifugation (7000*g,* 3 cycles). The peptide pellets were air-dried overnight at room temperature. Peptides were purified by reverse-phase HPLC with a Jupiter 4 μm Proteo C18 90 Å prep column, and HPLC fractions were examined to confirm by mass spectroscopy (microTO, Bruker Daltonics). Fractions containing the correct mass were pooled, lyophilized, and weighed to 0.1 μg accuracy using a Sartorius SE2 Ultra Micro Balance, then stored at −80 °C.

### Lipid Preparation for the Lipid-Induced Aggregation Method

Dry DMPS lipid powder was accurately weighed using a Sartorius ultramicro balance and dissolved in 20 mM sodium phosphate buffer (pH 6.5) to a final concentration of 2 mM. The lipid was solubilized by shaking in a 2 mL Eppendorf tube on a Thermomixer compact (Eppendorf) at 45 °C, 1400 rpm for 3 h. The solution was then freeze–thawed five times using dry ice and a thermomixer (45 °C, 500 rpm). Vesicle formation was completed by sonication, using a Bandelin Sonopuls sonicator equipped with a TS102 probe, set to 20% amplitude, for 15 cycles (30 s on/30 s off). Vesicles were freshly prepared on the day of use. The SUVs prepared have an average diameter of approximately 30–40 nm, as characterized by dynamic light scattering (DLS) as previously described.^24^ This corresponds to vesicle concentrations in the low micromolar range.

### Lipid-Induced Aggregation Assay

Lipid-induced aggregation experiments were monitored using a CLARIOstar fluorescence microplate reader (BMG Labtech) under quiescent conditions (no shaking) at 30 °C. Experiments were conducted in black, clear-bottom 96-well half-area polystyrene plates with a nonbinding surface (Corning #3881), sealed with aluminum Thermowell sealing tape (Corning #6570). Each well contained 100 μL of reaction volume, run in triplicate, comprising 100 μM αS, 50 μM ThT, 50 μM DMPS (monomeric equivalent), 0.01% sodium azide, and varying concentrations of peptide (0 μM to 1 mM), all in 20 mM phosphate buffer (pH 6.5).

Instrument settings included a focal height of 4.9 mm and a gain of 800, with an excitation filter of 440 ± 15 nm, an emission filter of 480 ± 15 nm, and a 460 nm dichroic cutoff. Readings were collected from the bottom optic using a spiral averaging (4 mm diameter) with 50 flashes per well, and measurements were taken every 1200 s. The outer wells of the plate were not used to avoid evaporation

### Agitation-Induced αS Aggregation Kinetic Assay

Aggregation induced by agitation assays were experiments were performed based on a protocol developed by Wordehoff et al.^50^ Briefly, the aggregation was monitored in a CLARIOstar fluorescence microplate reader (BMG Labtech), at 37 °C in black, clear-bottomed 96-well half-area polystyrene plates with Nonbonding surface (Corning #3881) covered with Aluminum Thermowell Sealing Tape (Corning #6570). The experiments were performed in 100 μL aliquots, in triplicate, each containing 100 μM αS, 20 μM ThT, 0.05% Sodium azide, and varying concentrations of the peptides (0 μM to 1 mM) in a buffer composed of 20 mM K_2_HPO_4_, 5 mM KH_2_PO_4_, and 100 mM KCl. A single glass bead of exactly 3 mm (VWR Cat. No. 201-1253) was added to each well to enhance mixing.

The focal height was set to 4.9 mm, and gain to 1000, with an excitation filter of 448–10 nm and emission filter of 482–10 nm and a dichroic cutoff of 465 nm. Well measurements were taken by spiral average of 4 mm using the bottom optic, with 12 flashes per well and a cycle time of 900 s. The plate was shaken in orbital mode at 400 rpm for 30 s before each cycle. The outer wells of the plate were not used to avoid evaporation.

### Transmission Electron Microscopy (TEM)

αS samples collected from the end point of the aggregation kinetic assay were prepared for TEM imaging. A 5 μL aliquot of each sample was applied to glow-discharged Formvar/carbon-coated 200 mesh copper grids for 1 min. Excess sample was removed with filter paper, and the grids were briefly washed twice with Milli-Q water (1 s each), blotting after each wash. The sample was stained by incubating the grids with 5 μL of uranyl acetate zero (Agar Scientific) for 30 s, followed by blotting to remove excess stain. Grids were air-dried for 2 h before imaging. Samples were visualized using a Jeol 2100 Plus transmission electron microscope operated at an accelerating voltage of 200 kV. Multiple grids were screened for each condition to obtain representative images of the samples.

### Circular Dichroism (CD) Spectroscopy

To measure the effect of DMPS SUVs on wild-type αS_1–140_ and αS_1–25_, 20 μM protein/peptide was incubated with increasing concentrations of DMPS SUVs (0–1.5 mM) in 20 mM phosphate buffer (pH 6.5) at 30 °C for 1 h. Far UV CD spectra scans were then recorded of the solutions on a Chirascan V100 (Applied Photophysics), at 30 °C, in a 1 mm path length quarts cuvette, scanning from 280–190 nm with a 1 nm bandwidth, averaged over 3 scans and blanked against the solutions containing the relevant DMPS vesicles in 20 mM phosphate buffer (pH 6.5).

Fractional helicity (fH) was calculated according to the equation

$$\mathrm{fH}=(\theta_{222}-\theta_{c})/(\theta_{222\infty }-\theta_{c})$$

where θ_222∞_ = (−44,000 + 250*T*)*­(1 – *k*/Nr) and θ_c_ = 2220 – 53*T*. In these equations, the wavelength-dependent constant *k* = 2.4, Nr = the number of residues, and *T* is the temperature, 30 °C.^68,69^

### PICUP Cross-Linking SDS-PAGE Electrophoresis

Photoinduced cross-linking of unmodified proteins (PICUP) was performed with modifications to a previously published protocol.^49^ Briefly, 20 μL of the endpoint (25 h) reaction mixture from the lipid-induced aggregation assay reaction mixture (100 μM αS, 50 μM ThT, 50 μM DMPS, 0–100 μM peptide in 20 mM sodium phosphate buffer, pH 6.5) was transferred to a 1.5 mL Eppendorf tube. To each sample, 2 μL of 1 mM solution of tris­(2,2’bipyridyl)­dichloro-ruthenium­(II) hexahydrate (Ru­(bpy)) and 2 μL of 20 mM ammonium persulfate (APS), both prepared in 20 mM sodium phosphate buffer (pH 6.5), were added simultaneously via a brief pulse in a desktop centrifuge. Samples were irradiated under ambient light for 10 s, then quenched with 10 μL of 4× RunBlue LDS Sample Buffer (Expedeon). After heating to 95 °C for 5 min, samples were resolved by SDS-PAGE using a 12% Tricine Gel. The protein bands were visualized with Instant Blue Coomassie stain (Expedeon).

### Peptide Stability in Human Serum

Serum stability assays were performed in normal human serum. Peptide stocks were initially prepared at 600 μM in H_2_O, and 75 μL of this stock was added to 1425 μL of normal human serum (Merck), yielding a final concentration of 30 μM. Samples were incubated at 37 °C, and 100 μL aliquots were collected at designated time points (0, 1, 2, 3, 4, 6, 22, 24, and 48 h) and then immediately stored at −80 °C. After all time points, the samples were thawed and mixed with 300 μL of acetonitrile:water (3:1, v/v) by vortexing to ensure complete mixing. Following centrifugation at 14500 rpm for 20 min, 200 μL of the supernatant was analyzed by HPLC using a Phenominex Luna 5 μm C18 100 Å analytical column, eluted with a 0–50% acetonitrile gradient containing 0.1% TFA. Peak areas were integrated and normalized to the 0-h sample. Each peptide was tested in triplicate.

### Nuclear Magnetic resonance (NMR) for the Peptide Structure

NMR spectroscopy was performed at 298 K on a Bruker Avance HD III 700 MHz spectrometer (Bruker, MA, USA) equipped with a 1.7 mm triple resonance TCI microcryoprobe using standard pulse sequences from the Bruker library. Samples contained unlabeled peptide at 1 mM in 20 mM phosphate buffer (pH 6.5) with 10% D_2_O and 50% TFE. Standard 2D NMR spectra (^1^H–^15^N HSQC, ^1^H^–13^C HSQC, ^1^H–^13^C-HSQC-TOCSY, ^1^H–^1^H TOCSY (80 ms), ^1^H–^1^H NOESY (150 and 250 ms) were acquired for resonance assignment. Data were processed using TopSpin 3.6.1 and analyzed with CCPNMR analysis v 2.4.2.^70^

Backbone dihedral angles were calculated from ^1^H_α_ and ^13^C_α/β_ chemical shifts using DANGLE,^71^ and combined with distance restraints extracted from the NOESY spectra. Structure calculations were performed using Aria v2.3.2 ^72^ coupled with CNS v1.21.^73,74^ Spin diffusion was enabled during the early refinement stages. In the final iteration, 200 structures were generated, and the 20 lowest-energy structures were further refined in water.

### Protein Expression of Uniformly ^15^N-Labeled Human wild-type αS_1–140_ for NMR studies

For NMR studies, uniformly ^15^N-labeled wild-type human αS was recombinantly expressed in *E. coli* T7 Express competent cells (New England BioLabs). Overnight cultures (100 mL) grown in M9 minimal medium supplemented with antibiotic (0.34 mg mL^–1^), ammonium sulfate (0.5 g L^–1^), and glucose (0.4%) were used to inoculate 1.6L of M9 similarly supplemented M9 minimal medium, in which ^15^NH_4_Cl (1 g L^–1^) was used as the sole nitrogen source. Cultures were grown at 37 °C with shaking (200 rpm) until an OD_600_ of 0.6 was reached, at which point protein expression was induced with 0.3 mM IPTG. After 4 h of further incubation, cells were harvested by centrifugation (6000 rpm, 30 min), flash frozen, and stored at −20 °C. Protein purification was performed as previously described.

### NMR of Lipid-Induced Aggregation

^1^H–^15^N TROSY experiments were performed at 303 K on a Bruker Avance III HD 700 MHz spectrometer equipped with a 1.7 mm triple-resonance TCI microcryoprobe using standard pulse sequences from the Bruker library. Two samples were prepared, each containing uniformly ^15^N-labeled αS (100 μM) and DMPS preformed into SUVs (100 μM) in 20 mM phosphate buffer (pH 6.5) with 10% D_2_O. Prior to acquisition, 100 μM αS_2–12_(L6) was added to one of the samples. ^1^H–^15^N TROSY spectra were acquired immediately after sample preparation and again following a 6-day incubation at room temperature.^70^

### SH-SY5Y Culture

Undifferentiated wild-type SH-SY5Y cells were maintained in T75 flasks in complete media: Dulbecco’s modified Eagle’s Medium/F-12 (Gibco, 11320033) supplemented with 10% fetal bovine serum, FBS (Life Technologies, 61965026), 100 Units mL^–1^ Penicillin-streptomycin (Life Technologies, 15140122), and l-glutamine (Life Technologies, 25030024). Cells were maintained at 37 °C and 5% CO_2_ and passaged using 0.25% trypsin-EDTA (Life Technologies, 25200056) when they reached 90% confluency, to a maximum of 15 passages.

### Peptide Uptake and Toxicity Assays

SH-SY5Y cells were plated at a density of 5,000 cells well^–1^ in a half-area 96-well plate. Twenty-four hours after plating, the media was removed and replaced with media containing 10 or 20 μM of rhodamine-B labeled peptide or the equivalent volume of PBS. Cells were incubated with peptides for a further 30–36 h. As a positive control for cell lysis, 3 wells of cells were treated with 5% Triton-X 100 for 20 min, and then 20 μL of media was harvested from all wells for ToxiLight analysis according to the manufacturer’s instructions (Lonza Biologics PLC, LT07–217). Cells were then washed once with PBS and incubated for 90 min with a 1:10 dilution of AlamarBlue reagent (Invitrogen, DAL1025). Media was harvested and the fluorescence measured according to the manufacturer’s instructions. Cells were then washed twice with PBS and incubated with a 1:12.5 dilution of NucBlue (ThermoFisher Scientific, R37605) and a 1:500 dilution of CellTracker Deep Red (Invitrogen, C34565) in media for a further 30 min. Cells were then washed 2 more times in PBS and imaged.

### Cell Assay Confocal Microscopy

Images were acquired using the Opera Phenix automated confocal microscope at 40× magnification over a Z-distance of 14 μm, taking optical slices at 1 μm intervals. Harmony software (PerkinElmer) was used for all image analysis.

### *C. elegans* Strain Production

The *C. elegans* strain N2 was used as the control, while the transgenic strain NL5901 (unc-54p::αS::YFP), which expresses αS fused to YFP in the body wall muscle cells, was used to model the PD motility phenotype. Worms were synchronized by bleaching with sodium hypochlorite, and the eggs were resuspended in M9 buffer and incubated overnight at 20 °C with shaking (50 rpm) to hatch. Larvae were then transferred to nematode growth medium (NGM) agar plates seeded with *E. coli* OP50 as a food source and incubated in the dark at 20 °C until they reached the L4 stage.

### *C. elegans* Thrashing Assay

L4-stage worms were transferred to fresh NGM agar plates containing 75 μM 5-fluoro-2’deoxyuridine (FUdR), to prevent reproduction, and seeded with *E. coli* OP50 as a food source. Plates were dosed with 2.2 mL aliquots of αS_2–12_(L6) peptide solution at final concentrations of 0, 25, 50, or 100 μM and then allowed to dry before the worms were added. Nematodes were incubated in the dark at 20 °C for 5 days. Following incubation, 15 worms per condition were picked using a worm pick and placed in a drop of M9 media on a microscope slide. Movements were recorded immediately for a period of 30 s. For each condition, 5 worms per video were manually scored for the number of body bends per minute. Worms that did not move were excluded. A body bend was defined as a full oscillation, where the head returned to a point on the same side.

### Inclusion Quantification in *C. elegans* by Fluorescence Microscopy

On adult Day 7, following the thrashing assay, worms received a second dose of αS_2–12_(L6) peptide at the same concentration and were incubated for a further 6 days. At least five worms from each condition were then transferred to individual wells of a black-walled 96-well plate, each containing 200 μL of M9 medium supplemented with 10 mM levamisole to induce paralysis.

Inclusions were initially visualized using confocal fluorescence microscopy on a Zeiss CellDiscoverer 7 LSM 900 equipped with an Airyscan detector (GaAsP-PMT) and a 5X Plan-Apochromat objective lens (NA 0.35). Imaging was performed with a 488 nm laser (0.8% power) using the following parameters: scan zoom of 1.3, scan speed of 7, sampling of 1.5, dwell time of 0.55 μs, gain of 700 V, and 2D Airyscan processing (version 8.1). Z-stacks with optimal subsampling were obtained for individual worms; however, laser illumination caused movement during confocal image acquisition even after anesthesia. Therefore, quantification was performed using single confocal slices per worm using Image. Bright spots (inclusions) and total YFP-positive areas were segmented by thresholding at 3000 AU (inclusions) and 100 AU (total YFP). The percentage of inclusion area (inclusion area/YFP area) and the mean areas of individual inclusions were calculated from the segmented masks.

To rapidly image whole nematodes and capture thick volumes, YFP epifluorescence and corresponding brightfield images were acquired using the Zeiss CellDiscoverer7 microscope in widefield mode with a 5x Plan-Apochromat objective lens (NA 0.35) and 0.5x Optivar (2.5x total magnification). YFP was imaged using a 470 nm LED (10% intensity, 100 ms exposure) with a 394/510/673 beam splitter and a 501–572 nm emission filter. YFP-positive worms and inclusions were automatically segmented using CellProfiler. Whole worms were identified based on YFP fluorescence, following the application of a Gaussian filter and primary object detection (global thresholding, Ostu two-class method, correction factor 5). Inclusions were segmented using a second Primary Object Detection step (20 μm window adaptive thresholding, minimum cross-entropy method, correction factor 2.5, typical object diameter 5–20 μm) and defined as tertiary objects within each worm. The number of inclusions per worm was quantified for 5 worms per condition.

## Supplementary Material



## Notes

Further detailed information relating to materials and methods utilized (PDF)
