# Supplementary material for: Stabilizing a Native Fold of Alpha-Synuclein with Short Helix-Constrained Peptides
Source: JACS Au. 2025 Sep 4;5(9):4321–36. doi: 10.1021/jacsau.5c00694 (PMC12458024; doi:10.1021/jacsau.5c00694)
Supplement: Supplementary file 1 [file au5c00694_si_001.pdf]

# Supporting Information for Publication

# Stabilising a Native Fold of Alpha-Synuclein with Short Helix-Constrained Peptides

Richard M. Meade[1*], Scott G. Allen[1], Amy Lopez [2], Christopher Williams[2], Iona Thomas-Wright[3], Rachel Heon-Roberts[3], Maria Carey Wood[1], T.M. Simon Tang[1], Julia E. Sero[1], Vicky L. Hunt[1], Richard Wade-Martins[3], Matthew P. Crump[2], Jody M. Mason[1]*

*[1]Department of Life Sciences, University of Bath, Claverton Down, Bath, BA2 7AY, United Kingdom*

*[2]School of Chemistry, University of Bristol, Cantock's Close, Bristol, BS8 1TS United Kingdom*

*[3]Oxford Parkinson's Disease Centre, Department of Physiology, Anatomy and Genetics, Oxford, OX1 3QX, United Kingdom*

**To whom correspondence should be addressed: j.mason@bath.ac.uk or rm288@bath.ac.uk*

Keywords: peptide, amyloid aggregation, lipid induced aggregation, lipid vesicles, Parkinson's disease

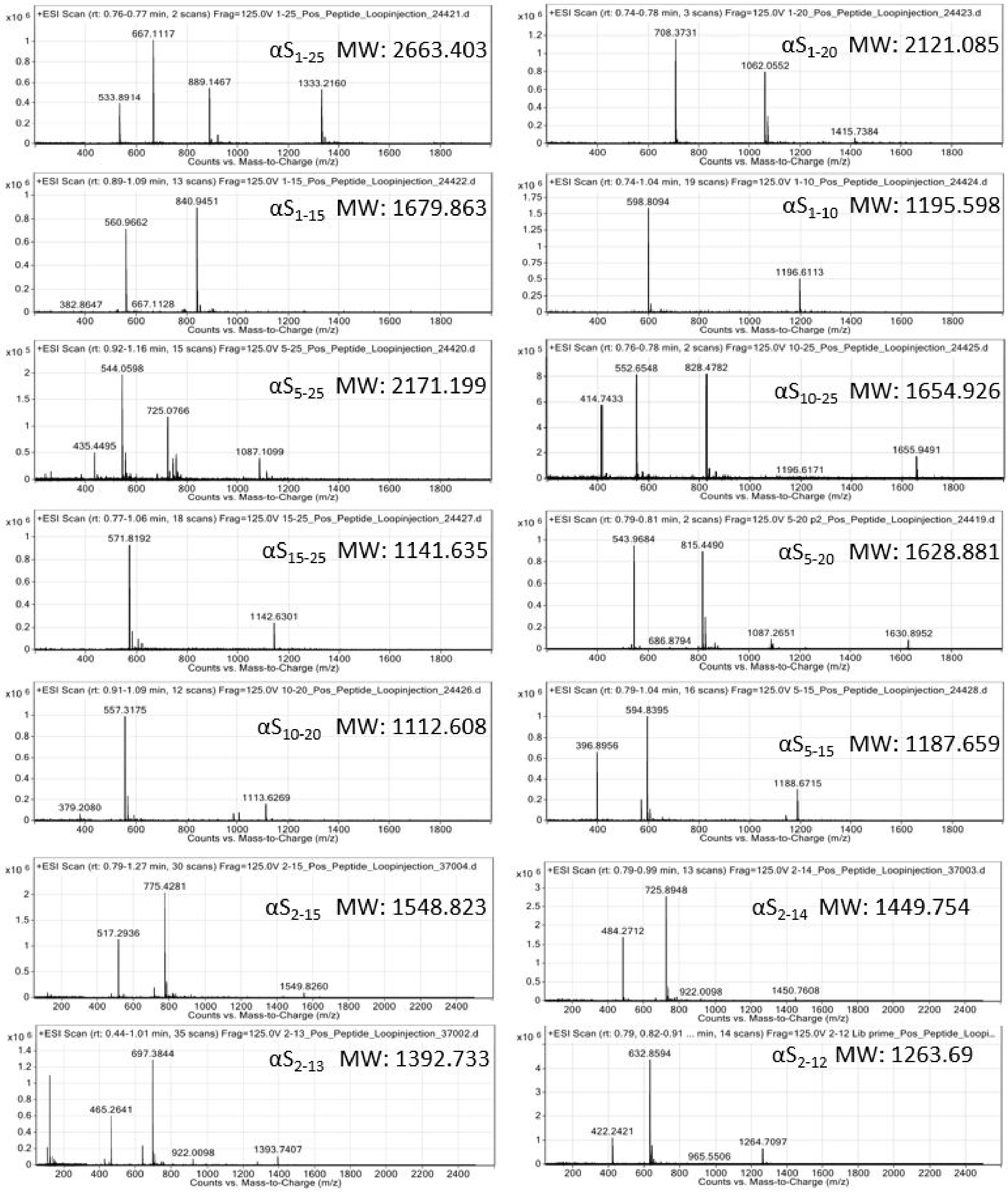


***Supplementary Figure 1: Mass spectra of the purified linear peptides.***

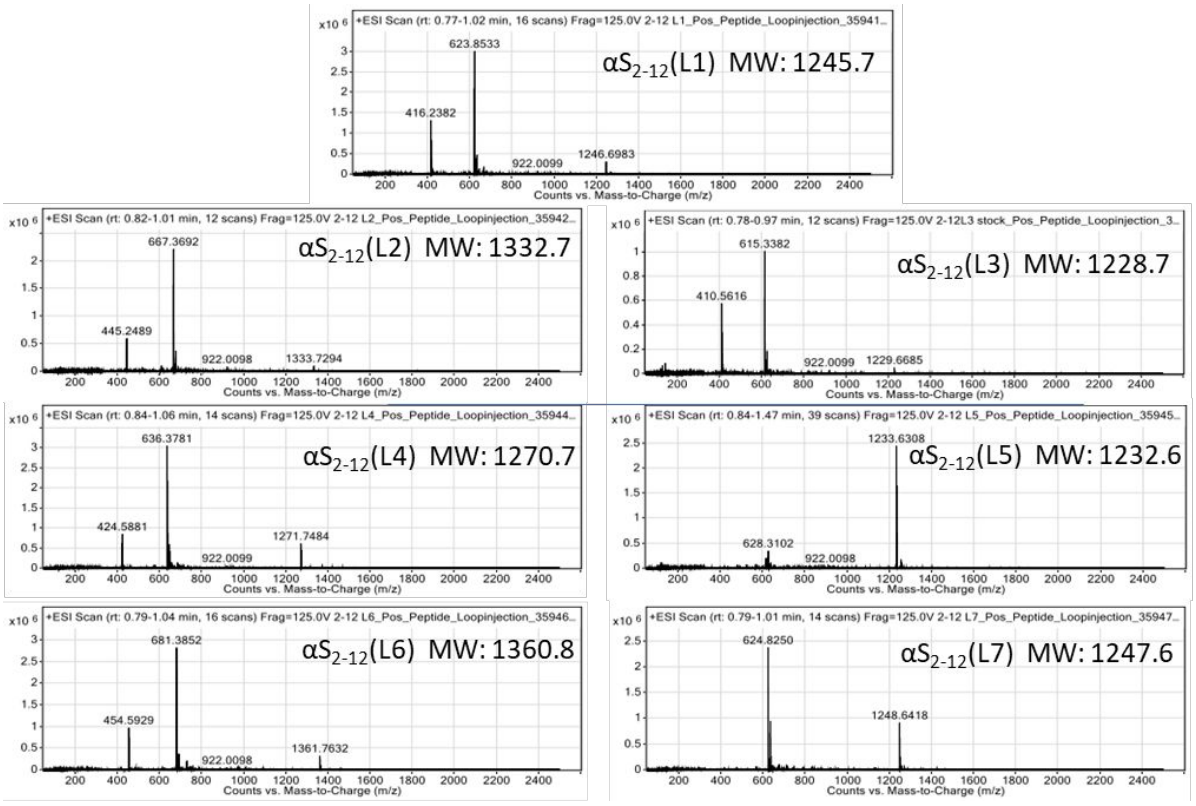


***Supplementary Figure 2: Mass spectra of the purified lactam constrained peptides.***

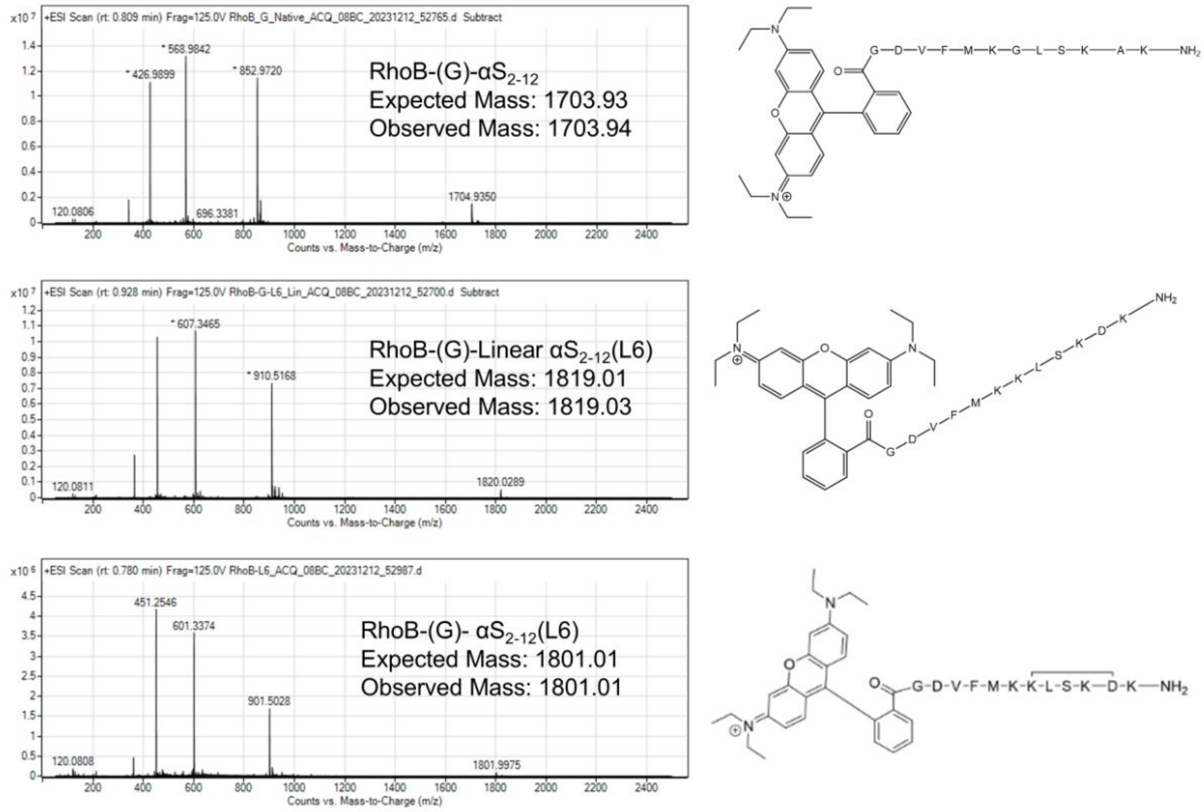


***Supplementary Figure 3: Mass spectra of the purified lactam constrained and RhodamineB-labelled peptides.***

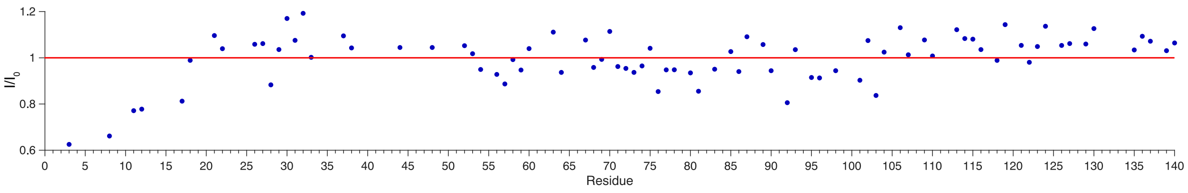


***Supplementary Figure 4:*** *A plot of the relative $^1H$ -$^{15}N$ HSQC peak intensities between the sample with $\alpha S_{2\text{-}12}L6$ on day 6 (I) and day 0 ($I_0$) for each assignable residue. The majority of peaks have a ratio of approximately one (red line at y = 1) indicating that monomeric αS has been preserved over the six-day incubation period.*

## Displacement of αS from DMPS lipid vesicles

Displacement of αS from DMPS vesicles by $\alpha S_{2\text{-}12}$ and $\alpha S_{2\text{-}12}$(L6) was monitored by $^1H$-$^{15}N$ TROSY-HSQC experiments carried out on a Bruker Avance III HD 700 MHz spectrometer equipped with a 1.7 mm inverse triple-resonance micro-cryocoil probe. Experiments were performed at 303 K using the standard best TROSY pulse sequence from the Bruker library. Spectra were referenced to $H_2O$, and the sweep widths used were 9 ppm and 25 ppm for $^1H$ and $^{15}N$ respectively. Backbone $^1H$-$^{15}N$ chemical shift assignments could be made by reference to published data on αS in the free, unstructured form and a control experiment without lipid vesicles of $^{15}N$-labelled αS (30 µM) in NaP (20 mM), pH 6.5 with 10% $D_2O$. For titration experiments, samples contained $^{15}N$-labelled αS (20-30 µM) and DMPS pre-formed into SUVs (1.5 mM) in NaP (20 mM), pH 6.5 with 10% $D_2O$. Peptide samples were prepared in the same manner with the addition of the relevant peptide. For $\alpha S_{2\text{-}12}$(L6), samples were prepared with a 10-fold, 15-fold and 20-fold molar excess of peptide. For the linear $\alpha S_{2\text{-}12}$, a 15-fold molar excess was used.

## Displacement of αS from DMPS lipid vesicles Methods

A pair of 2D $^1H$-$^{15}N$ TROSY-HSQC NMR experiments were recorded to monitor lipid interactions of $\alpha S_{2\text{-}12}$(L6) and linear control $\alpha S_{2\text{-}12}$ with αS. $^1H$-$^{15}N$ TROSY-HSQC spectra were acquired at a [DMPS]:[αS] ratio of 50, whereby all αS would be in the lipid-bound form. Amide cross peaks of αS (30 µM) with DMPS (1.5 mM) had intensity ratios of bound ($I$) to free form ($I_0$) of approximately 0.6 for residues 3-60, while the C-terminal residues had ratios of approximately 0.8, in line with immobilisation of the N-terminal helical region and retention of a dynamic C-terminal (Figure S5A, S5B). The remainder of the residues (60-110) had intermediate intensity ratios. Upon addition of a 15-fold molar excess of $\alpha S_{2\text{-}12}$(L6), peak intensities for residues across the protein increased, with the largest increases observed in the N-terminus and NAC region (Figure S5B). This increase in intensity suggested that $\alpha S_{2\text{-}12}$(L6)

modifies the lipid binding of $aS_{1\text{-}140}$. Even at this excess molar ratio however, there is evidently persistent exchange from free to bound form as ($I/I_0$) remains < 1 for the majority of N-terminal residues. Interestingly, the same effect was not observed upon addition of $\alpha S_{2\text{-}12}$ (Figure S5C), implying that this linear peptide had no effect on lipid binding of αS and the mechanism is unlikely to be driven simply by the peptide occluding αS lipid binding via non-specific lipid interactions. Combined with the observations that a monomeric αS species was preserved throughout the aggregation assays, one possible mechanism is that lipid-induced primary nucleation by $\alpha S_{2\text{-}12}$(L6) aggregation may be prevented by impeding the formation of the aggregation-prone lipid-induced structure of αS, possibly through perturbed lipid binding.

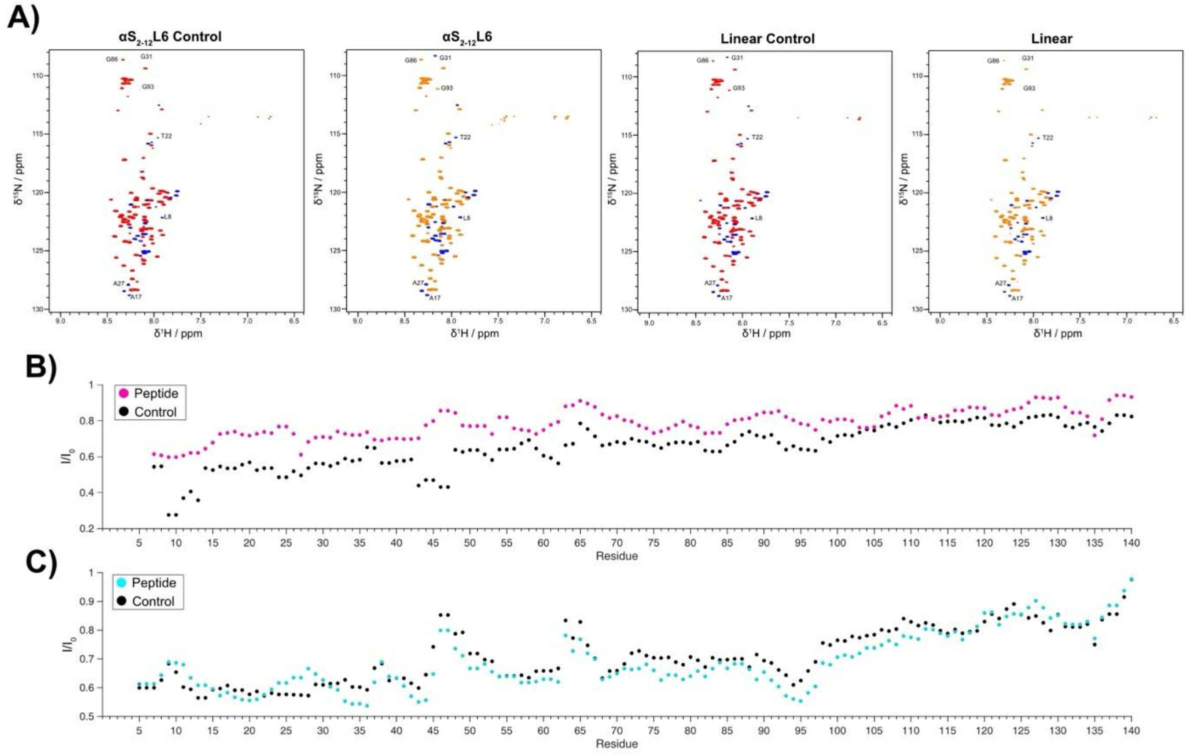


***Supplementary Figure 5.*** ***(A)*** *$^{1}H$-$^{15}N$ TROSY-HSQC spectra of the control (red) and peptide (yellow) samples recorded at pH 6.5, 303 K. N-terminal residues from 1-60 are highlighted in blue with residues showing the largest intensity changes upon addition of $\alpha S_{2\text{-}12}$(L6) labelled.* ***(B)*** *Relative peak intensities of amide peaks in the HSQC spectrum of αS plus DMPS without $\alpha S_{2\text{-}12}$ (L6) (black) compared to a sample of free αS and with a 15-fold molar excess of $\alpha S_{2\text{-}12}$ (L6) (magenta) plotted as a rolling average over a 5-residue window.* ***(C)*** *Relative peak intensities of amide peaks in the HSQC spectrum without $\alpha S_{2\text{-}12}$ (L6) (black) compared to a sample of free αS and with a 15-fold molar excess of $\alpha S_{2\text{-}12}$linear (without the lactam linker in place) (blue) plotted as a rolling average over a 5-residue window.*

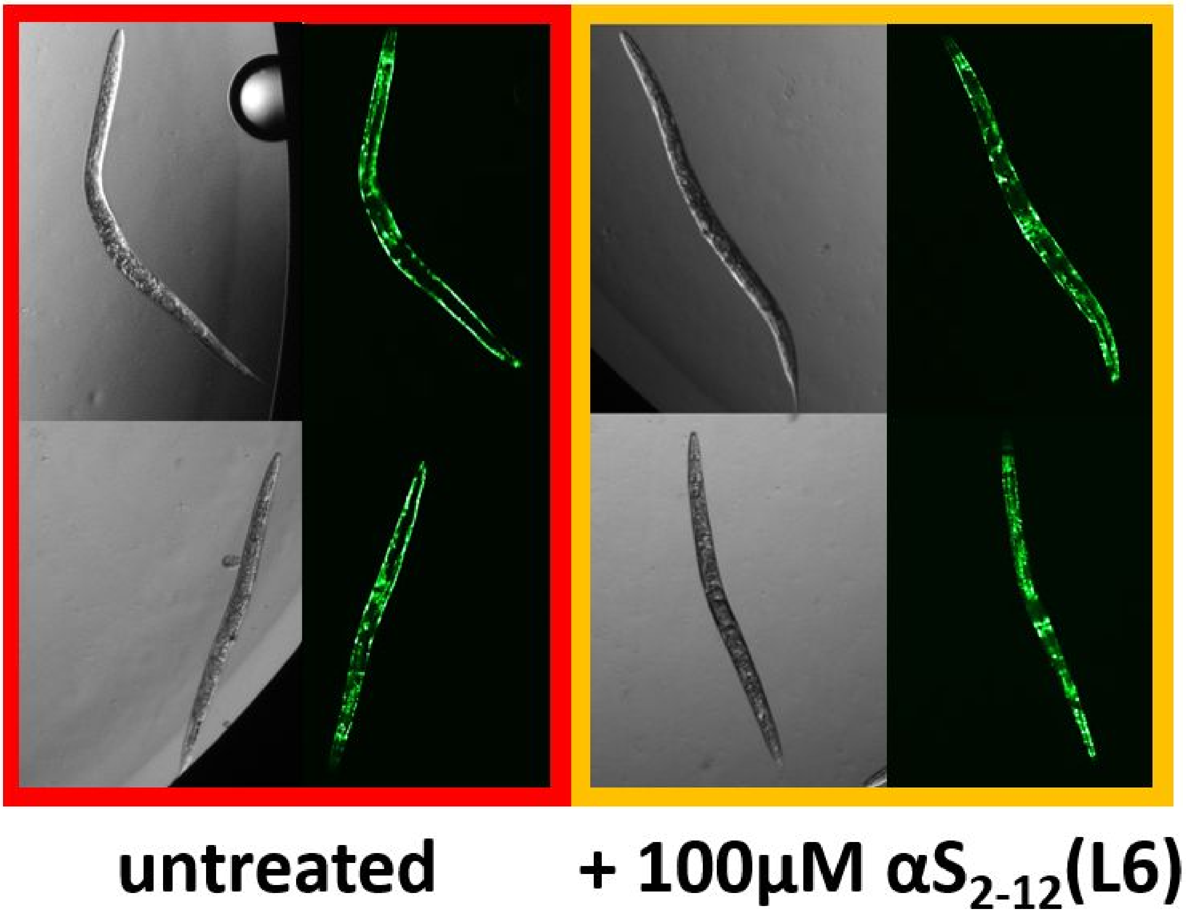


***Supplementary Figure 6:*** *Representative full-body images of showing the effects of αS$_{2-12}$(L6) on the formation of inclusions in the worms expressing αS-YFP.*

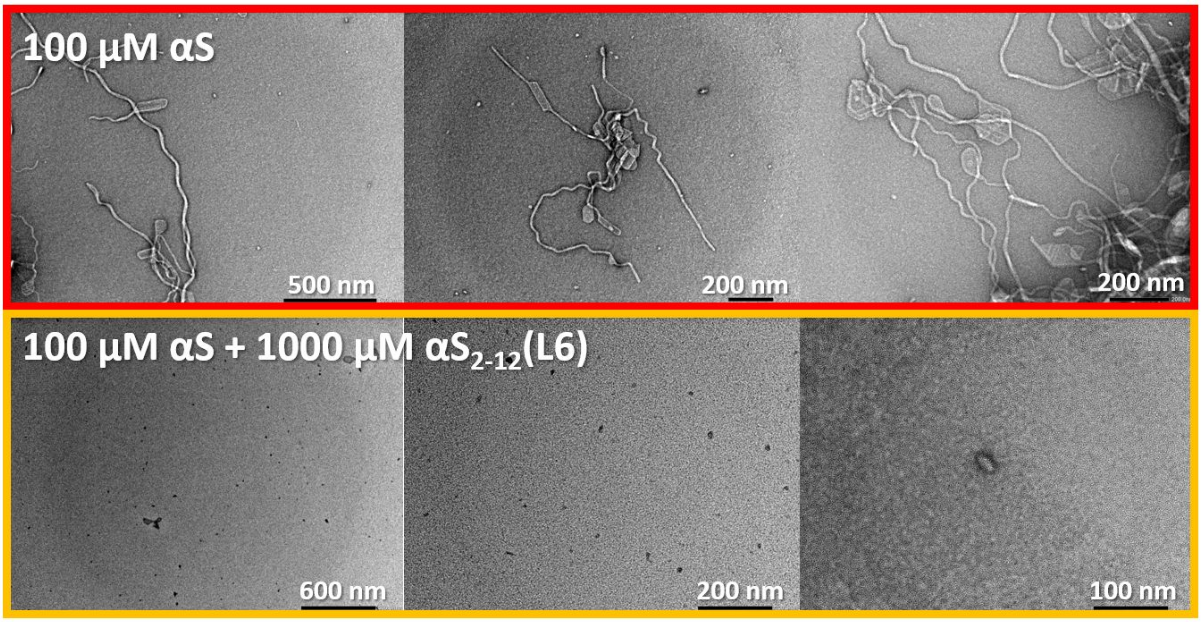


***Supplementary Figure 7:*** *TEM of lipid induced aggregation repeat clearly showing lipid induced fibrils in the αS control, which are notably absent in the presence of αS$_{2-12}$(L6).*

| Parameter | $\alpha S_{2\text{-}12}$(L6) |
|---|---|
| **PDB ID** | 8OL8 |
| **Degree of Assignment**[1] | |
| H (%) | 97.4 |
| Heavy atoms (N & C) (%) | 72.6 |
| **Number of Restraints** | |
| Intra-residue (\|i-j\|=0) | 66 |
| Sequential (\|i-j\|=1) | 35 |
| Medium Range (2 ≤ \|i-j\| < 5) | 20 |
| Long Range (\|i-j\| ≥ 5) | 0 |
| Ambiguous | 16 |
| Total | 137 |
| Dihedral Angle (ϕ/ψ) | 14 |
| **Restraint Statistics**[2] | |
| RMSD of distance violations (Å) | 0.032 (±0.012) |
| RMSD of dihedral violations (°) | 0.013 (±0.048) |
| Violations >0.5 Å | 0.00 (±0.00) |
| Violations >0.3 Å | 0.50 (±0.74) |
| Violations >0.1 Å | 3.00 (±1.05) |
| **RMSD from Idealised Covalent Geometry**[2] | |
| Bonds (Å) | 0.003 (±0.0001) |
| Angles (°) | 043 (±0.02) |
| Impropers (°) | 0.86 (±0.12) |
| **Structural Quality** | |
| Ramachandran Statistics[3] | |
| Most Favoured Regions (%) | 99.4 |
| Allowed Regions (%) | 0.6 |
| Generously Allowed Regions (%) | 0.0 |
| Disallowed Regions | 0.0 |
| Verify3D Z-score[2] | -8.03 |
| Prosa II Z-score[2] | -4.63 |
| Procheck Z-score (Backbone)[2] | 1.10 |
| Procheck Z-score (Dihedral)[2] | 0.06 |
| MolProbity Z-score[2] | 0.47 |
| Number of Clashes[2] | 0 |
| **Co-ordinates Precision (RMSD)**[1] | |
| Secondary Structure Backbone Atoms (Å) | 0.13 (±0.04) |
| Secondary Structure Heavy Atoms (Å) | 0.54 (±0.13) |
| All Backbone Residues (Å) | 0.61 (±0.14) |
| All Heavy Atoms (Å) | 1.05 (±0.24) |

1. cns v1.21
2. PDB Validation v8.061
3. Procheck v3.5.4, residues 3-10.

**Supplementary Table 1.** NMR Assignment, Structure Calculation and Validation Statistics
